# Blood pressure variability and cardiovascular disease: systematic review and meta-analysis

**DOI:** 10.1136/bmj.i4098

**Published:** 2016-08-09

**Authors:** Sarah L Stevens, Sally Wood, Constantinos Koshiaris, Kathryn Law, Paul Glasziou, Richard J Stevens, Richard J McManus

**Affiliations:** 1 Nuffield Department of Primary Care Health Sciences, University of Oxford, Radcliffe Observatory Quarter, Oxford OX2 6GG, UK; 2 Faculty of Health Sciences and Medicine, Bond University, Queensland, Australia

## Abstract

**Objective** To systematically review studies quantifying the associations of long term (clinic), mid-term (home), and short term (ambulatory) variability in blood pressure, independent of mean blood pressure, with cardiovascular disease events and mortality.

**Data sources** Medline, Embase, Cinahl, and Web of Science, searched to 15 February 2016 for full text articles in English.

**Eligibility criteria for study selection** Prospective cohort studies or clinical trials in adults, except those in patients receiving haemodialysis, where the condition may directly impact blood pressure variability. Standardised hazard ratios were extracted and, if there was little risk of confounding, combined using random effects meta-analysis in main analyses. Outcomes included all cause and cardiovascular disease mortality and cardiovascular disease events. Measures of variability included standard deviation, coefficient of variation, variation independent of mean, and average real variability, but not night dipping or day-night variation.

**Results** 41 papers representing 19 observational cohort studies and 17 clinical trial cohorts, comprising 46 separate analyses were identified. Long term variability in blood pressure was studied in 24 papers, mid-term in four, and short-term in 15 (two studied both long term and short term variability). Results from 23 analyses were excluded from main analyses owing to high risks of confounding. Increased long term variability in systolic blood pressure was associated with risk of all cause mortality (hazard ratio 1.15, 95% confidence interval 1.09 to 1.22), cardiovascular disease mortality (1.18, 1.09 to 1.28), cardiovascular disease events (1.18, 1.07 to 1.30), coronary heart disease (1.10, 1.04 to 1.16), and stroke (1.15, 1.04 to 1.27). Increased mid-term and short term variability in daytime systolic blood pressure were also associated with all cause mortality (1.15, 1.06 to 1.26 and 1.10, 1.04 to 1.16, respectively).

**Conclusions** Long term variability in blood pressure is associated with cardiovascular and mortality outcomes, over and above the effect of mean blood pressure. Associations are similar in magnitude to those of cholesterol measures with cardiovascular disease. Limited data for mid-term and short term variability showed similar associations. Future work should focus on the clinical implications of assessment of variability in blood pressure and avoid the common confounding pitfalls observed to date.

**Systematic review registration** PROSPERO CRD42014015695.

## Introduction

Blood pressure is a leading risk factor for cardiovascular disease.^1^ ^2^ Most studies have used mean blood pressure as the indicator of risk, measured in clinic or “out of office” settings.^3^ ^4^ ^5^ However, blood pressure shows noticeable oscillations over the short and long term.^6^ Historically, variability in blood pressure has been viewed as inhibiting accurate measurement of mean blood pressure and as a phenomenon to be overcome by improved monitoring.^7^ For at least two decades, this variability has also been recognised as a potential risk factor in its own right.^8^ ^9^ In 2010 an analysis of three cohort studies and two randomised trials found that long term variability in blood pressure was a predictor of stroke and coronary events in high risk patients.^10^

However, understanding this variability has been hampered by statistical and clinical methodological problems. Some analyses of variability have not adjusted for mean blood pressure, potentially confounding high variability with high mean blood pressure,^11^ or have adjusted for a mean that is not fully consistent with the variability measure.^12^ Others, in using 24 hour mean to adjust for daytime variability, might have turned high daytime variability into a surrogate marker for nocturnal or 24 hour blood pressure.^13^ Further studies have defined variability on the basis of measurements taken during follow-up, but analysed it as a baseline risk factor,^14^ ^15^ ^16^ potentially introducing problems of informative censoring or immortal time bias.^17^ Informative censoring occurs when reasons for loss to follow-up are confounded with the exposure (eg, if individuals with extreme or erratic blood pressures are withdrawn from studies because of concerns about safety). Immortal time bias can occur if individuals are required to have a certain number of blood pressure measurements in order to be included in analysis for mortality outcomes. The time up until the qualifying measurement becomes “immortal time,” because, by definition, death could not occur earlier.

Other studies failed to use consistent blood pressure monitoring equipment over time, to define a consistent measurement protocol, or to account for change in drugs, leaving doubt as to the source of any observed variability.^14^ ^18^ ^19^ Measurement at different times of the day^20^ or year,^21^ in different arms,^22^ or using inconsistent cuff sizes^23^ can affect accurate measurement, thereby inducing variability. We reviewed prospective studies in adults that quantified the associations of blood pressure variability with cardiovascular events and mortality, independent of mean blood pressure. Our main analysis focused on studies meeting prespecified methodological criteria, so that any apparent effect of variability was likely to be a true independent effect.

## Methods

### Study selection

We searched Medline, Embase, Cinahl, and Web of Science to 15 February 2016 for full text articles in English describing trials and prospective cohort studies in adults that assessed the association of periods of variability in blood pressure with cardiovascular outcomes (see supplementary table e1). Long term variability was measured through clinic blood pressure monitoring, mid-term through home monitoring, and short term through ambulatory monitoring. Studies included in recent systematic reviews^24^ ^25^ ^26^ ^27^ were also screened. Two reviewers (SW/SS and KL/KC) scrutinised the titles and abstracts, with adjudication by a third reviewer (RM).

### Inclusion and exclusion criteria

Studies had to consider at least one of the following outcomes: all cause mortality, cardiovascular events (including stroke, myocardial infarction, coronary heart disease, and heart failure), or cardiovascular mortality (including sudden death). We excluded studies only assessing intermediate outcomes (eg, arterial intima media thickness) or concerning nocturnal dipping or day-night variation, as these have been considered previously.^28^

Studies in disease specific populations (eg, people with diabetes) were included, except those in patients receiving haemodialysis where changes in blood pressure (intradialysis hypotension and hypertension^29^ ^30^) are common and have been shown to be associated with hospital admission and mortality.^31^ ^32^

Included studies had at least 2500 person years of follow-up. Blood pressure variability was assessed in the long term (in clinics), mid-term (at home), or short term (through ambulatory monitoring). Studies of clinic monitoring had to measure visit-to-visit variability over at least five clinic visits. Studies of home monitoring had to consider day-by-day variability over at least 12 measurements on at least three days.^33^ Studies for ambulatory monitoring had to assess variability up to 24 hours, with at least 14 daytime readings.^33^

### Data extraction

Using prespecified forms, two reviewers (SS/SW and KL/RM) independently extracted data on study and patient characteristics and two (SS and KC/RS) on statistical results (see supplementary table e2). Hazard ratios were extracted for every variability measure and outcome. The hazard ratio from the analysis with the greatest adjustment for confounders but containing only a single variability measure was extracted. Where required data were not available, we emailed the study authors.

### Data analysis and statistical methods

Hazard ratios were converted to standardised hazard ratios, using a general method for regression models (see supplementary table e3).^34^ Briefly, a standardised log-hazard ratio was calculated as the log-hazard ratio for each unit of standardised blood pressure variability (blood pressure variability divided by its sample standard deviation). These were pooled using a random effects meta-analysis, stratified by outcome. Separate analyses were performed for each period of variability (long term, mid-term, or short term). Heterogeneity was assessed using the χ^2^ test and I^2^ statistic.

Where studies used multiple measures of variability, we included hazard ratios in analysis according to the following hierarchy (preferred to least preferred): standard deviation, coefficient of variation, variation independent of mean, average real variability, standardised residual, root successive variance, and other. Where hazard ratios were calculated using data from the same primary study but reported in different papers, we included the most recently published hazard ratio. We combined the hazard ratios for study subgroups before inclusion.

Two reviewers (SS and RS) independently assessed the risk of bias using the QUIPS tool,^35^ with adjudication by a third reviewer (RM). We also extracted information about other potential confounders, specific to studies of blood pressure variability (see supplementary table e2). Consistency of blood pressure measurement with respect to device, cuff size, staff, and measurement is important to prevent inducing the variability. The impact of other potential confounders may be adjusted for during analyses. We decided (a priori) to include in main analyses only hazard ratios that were correctly adjusted for the equivalent mean blood pressure level (eg, adjusted for mean daytime systolic blood pressure if variability was assessed for daytime systolic blood pressure), where outcome ascertainment took place after the blood pressure measurement period and, for studies involving antihypertensive treatment, where at least 80% of patients were adherent to treatment or did not change drugs during the measurement period, or where patients were censored at the point of change of treatment. We carried out secondary analyses including all studies.

Publication bias was assessed by Egger’s test.^36^ However, since this has low power for small numbers of studies, we also calculated the number of null effect studies of mean weight that would need to be included in meta-analyses to result in a non-significant pooled effect (known as fail-safe N).^37^

### Patient involvement

Two lay representatives contributed to the design and content of the National Institute for Health Research programme grant from which this work arose. Results from this work have been presented as part of the wider programme at regular steering group meetings.

## Results

Searches identified 5861 references. Removal of duplicates and screening by two reviewers yielded 41 full text articles for inclusion (fig 1). These 41 papers represented 19 observational cohort studies and 17 clinical trial cohorts, and 46 separate analyses (see supplementary table e4). Twenty four papers^10^ ^14^ ^15^ ^16^ ^18^ ^19^ ^38^ ^39^ ^40^ ^41^ ^42^ ^43^ ^44^ ^45^ ^46^ ^47^ ^48^ ^49^ ^50^ ^51^ ^52^ ^53^ ^54^ ^55^ studied long term variability (ie, monitoring of blood pressure in clinics), four^56^ ^57^ ^58^ ^59^ studied mid-term variability (home monitoring), and 15^10^ ^11^ ^12^ ^13^ ^41^ ^60^ ^61^ ^62^ ^63^ ^64^ ^65^ ^66^ ^67^ ^68^ ^69^ studied short term variability (ambulatory monitoring). The number of participants in each study ranged from 457^41^ to 122 636^54^ and follow-up ranged from 2514 person years^41^ to 490 544 person years.^54^

**Figure f1:**
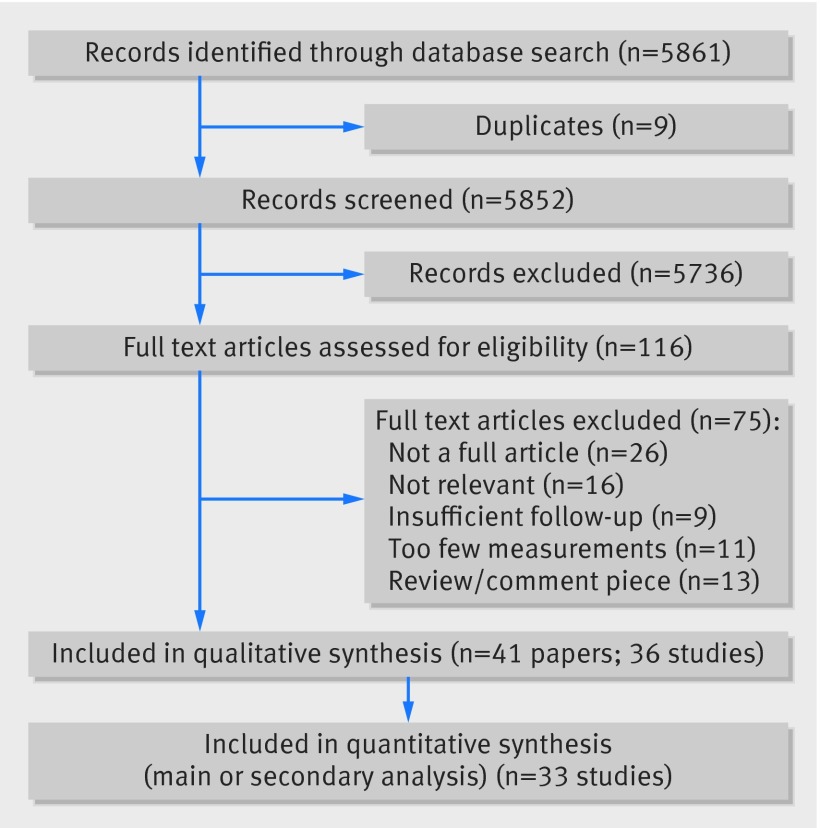
**Fig 1** Study screening flowchart

### Study design and analysis characteristics

Consistency of blood pressure measurement with respect to cuff size, arm, device, and staff was unclear or had the potential to introduce variability (eg, mercury sphygmomanometers and changing staff) in all of the 36 included studies (see supplementary table e5). Similarly, the potential for confounding was introduced because of the analysis (or this was unclear) in all 46 separate analyses. Results from 23 analyses were excluded from our main analyses on the basis of the three prespecified criteria: eight analyses failed to correctly adjust for mean blood pressure, 15 did not account for major drug change during the measurement period, and 20 did not separate the measurement and follow-up periods. Results from four analyses (three studies) were not reported in sufficient detail to allow data extraction.

### QUIPS risk of bias

Using QUIPS, most of the 46 analyses were rated at moderate risk of bias for study participation, often because of inclusion criteria based on blood pressure readings and a potential for regression to the mean effects (see supplementary table e6). Eighteen analyses were at high risk of bias because the measurement period for blood pressure variability was confounded by follow-up (n=17), and one analysis^67^ failed to report non-significant results. All of the analyses rated at high risk of bias using QUIPS were excluded from our main analysis based on the assessments in supplementary table e5.

#### Long term variability measured by clinic monitoring

Twenty four papers reported results from 27 studies that measured blood pressure variability in clinics (long term). Results from three studies^44^ ^48^ ^52^ were not presented in sufficient detail for extraction.

Eight studies examined long term variability in systolic blood pressure and all cause mortality, of which four had sufficiently low risk of bias to be included in the main analysis (fig 2, standardised hazard ratio 1.15, 95% confidence interval 1.09 to 1.22). Heterogeneity between studies (I^2^=70.7%, P=0.02) was reduced after removal of a study in patients with previous stroke or vascular disease^43^: this did not significantly alter the results (hazard ratio 1.12, 95% confidence interval 1.08 to 1.16; I^2^=34.9%, P=0.21).

**Figure f2:**
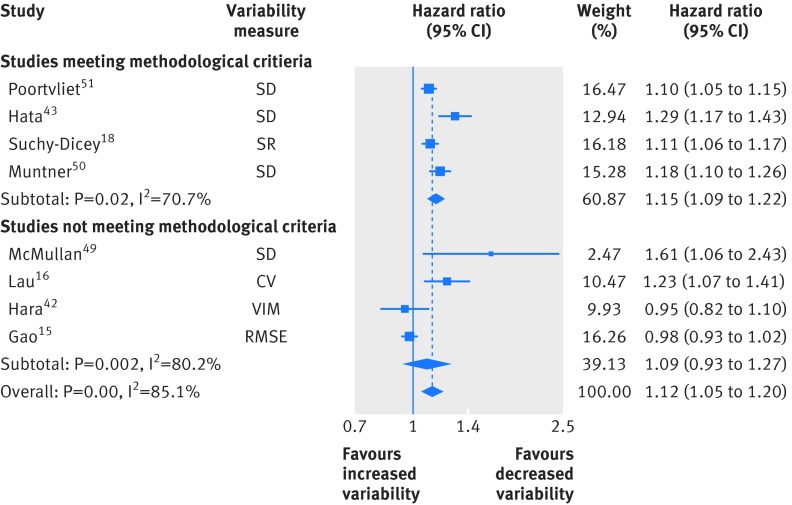
**Fig 2** Random effects meta-analysis of standardised hazard ratios for increases in clinic systolic blood pressure variability and all cause mortality. SD=standard deviation; SR=standardised residual; CV=coefficient of variation; VIM=variation independent of the mean; RMSE=root mean squared error

Three studies assessing blood pressure variability and cardiovascular disease mortality showed a significant relation (see supplementary figure e1, hazard ratio 1.18, 95% confidence interval 1.09 to 1.28) but only a single study examining cardiovascular disease events was suitable for inclusion (see supplementary figure e2, 1.18, 1.07 to 1.30).

Fourteen studies reported results for stroke events, of which six were included in the main analysis (fig 3; 1.15, 1.04 to 1.27; I^2^=82.1%, P<0.001). Results were similar after omission of the hazard ratio from the UK-TIA trial,^70^ which removed the heterogeneity (1.10, 1.05 to 1.14; I^2^=0.0%, P=0.62).

**Figure f3:**
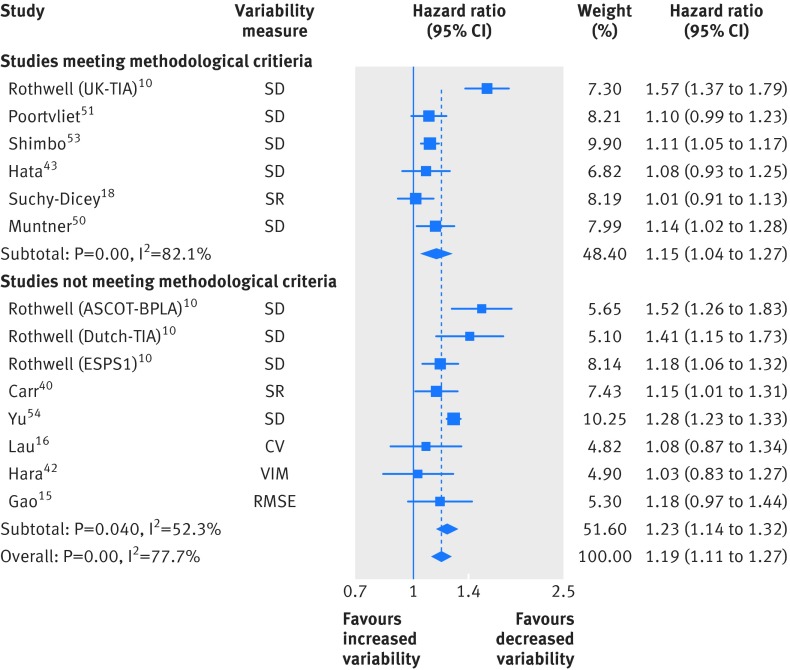
**Fig 3** Random effects meta-analysis of standardised hazard ratios for increases in clinic systolic blood pressure variability and stroke events. SD=standard deviation; SR=standardised residual; CV=coefficient of variation; VIM=variation independent of the mean; RMSE=root mean squared error

Results for coronary heart disease events and myocardial infarction showed similar results (see supplementary figures e3 and e4). Across all outcomes, secondary analysis including results from all studies regardless of risk of bias did not alter results.

#### Mid-term variability measured by home monitoring

Four papers reported results from two studies that measured mid-term variability in home blood pressure monitoring. All four papers were of sufficient quality to be included in main analyses, but a lack of data from distinct studies meant it was only possible to perform formal meta-analysis for the all cause mortality outcome. Variability in systolic blood pressure was a significant predictor of death when blood pressure was measured in the morning or evening, or both (fig 4, eg, hazard ratio for increases in combined blood pressure variability 1.15, 95% confidence interval 1.06 to 1.26). Study level results for other outcomes are given in supplementary table e7).

**Figure f4:**
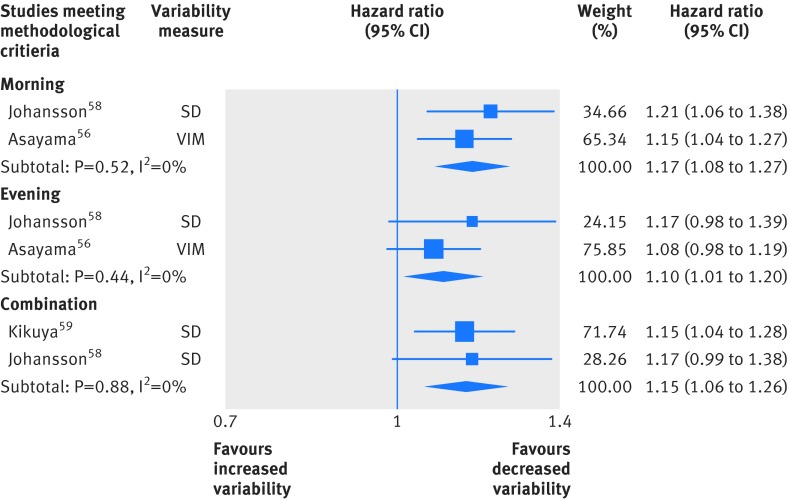
**Fig 4** Random effects meta-analysis of standardized hazard ratios for increases in home systolic blood pressure variability and all cause mortality. SD=standard deviation; VIM=variation independent of mean

#### Short term variability measured by ambulatory monitoring

Fifteen papers examined short term variability in ambulatory blood pressure in 11 distinct studies. We were unable to include results from many studies,^11^ ^13^ ^56^ ^57^ ^41^ ^69^ ^65^ ^66^ ^67^ owing to overlap with two large studies (IDACO^61^ and ABP-International^64^), which combined results across cohorts.

Three studies examined daytime variability in systolic blood pressure and all cause mortality, of which two were included in the main analysis (fig 5; hazard ratio 1.10, 95% confidence interval 1.04 to 1.16). Four studies examined daytime variability in blood pressure and cardiovascular disease mortality, and analysis of three studies with low risk of bias showed a significant association (see supplementary figure e5, 1.12, 1.03 to 1.21). Daytime blood pressure variability was also significantly associated with increased risk of stroke (see supplementary figure e6; 1.11, 1.01 to 1.21). Results for all three outcomes were unchanged in secondary analysis including results from all studies.

**Figure f5:**
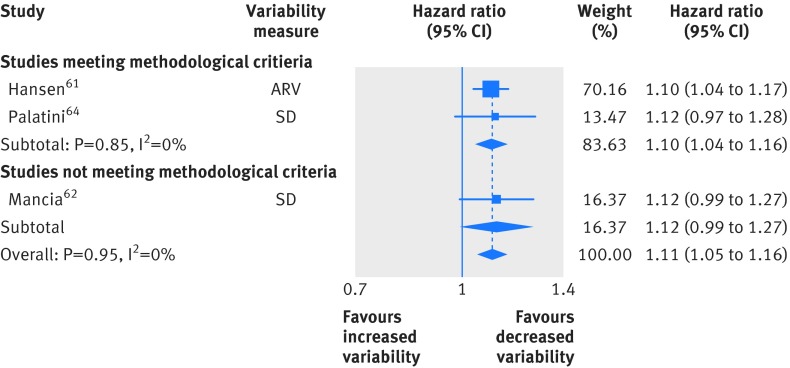
**Fig 5** Random effects meta-analysis of standardised hazard ratios for increases in variability of ambulatory systolic blood pressure and all cause mortality. SD=standard deviation; ARV=average real variability

No associations were found between variability in blood pressure and cardiovascular disease (see supplementary figure e7) or coronary heart disease events (see supplementary figure e8), although results became significant in secondary analyses. The supplementary file details the results for night-time and 24 hour systolic ambulatory blood pressure (see figures e9 to e18).

### Publication bias

There was no evidence of publication bias for any outcome in relation to long term, mid-term, or short term variability in systolic blood pressure as judged by Egger’s test. Significant findings for clinic monitoring would remain unchanged for all outcomes even if at least 20 null effect studies were included in meta-analyses, except for myocardial infarction where only a single null effect study would be required. Results for home monitoring would become non-significant after the addition of a single null effect study and those for variability in ambulatory blood pressure would become non-significant by the addition of between one and six null effect studies, depending on outcome and period of measurement.

## Discussion

This review has systematically assessed the literature for the association of long term (clinic monitoring), mid-term (home monitoring), and short term (ambulatory) variability in blood pressure with cardiovascular outcomes and mortality. Long term variability in measurements is significantly associated with all cause and cardiovascular disease mortality, cardiovascular disease events, stroke, and myocardial infarction, independent of mean blood pressure. Mid-term and short term variability are also associated with mortality, and limited data for other outcomes also broadly support an association with cardiovascular outcomes. Across all analyses (long term, mid-term, and short term), the hazard ratios for coronary heart disease events were smaller than those for stroke, suggesting that the effect observed for cardiovascular disease events—as with mean blood pressure—may be driven primarily by cerebrovascular events.

### Strengths in relation to the literature

This review includes over one million person years of data and combines the results from long, mid-term, and short-term blood pressure measurement, allowing comparison. We have addressed the methodological issues that are particular to research on variability in blood pressure and have shown that although there is now considerable evidence on this topic, most studies are of poor quality or poorly reported. By limiting our main analysis to studies that avoid potential sources of confounding, this review confirms that the apparent prognostic value of blood pressure variability is a true prospective association and can be demonstrated even in studies with low risk of bias.

In this review we used standardised hazard ratios to overcome the diversity of measures for variability used in primary studies, and hence combined more data. For example, our meta-analysis for long term variability and stroke events includes 14 studies, more than double the number in previous analyses.^24^ ^25^ This review also had sufficient data for meta-analysis of the effect of short term variability of blood pressure on outcomes, which was a limitation of a previous work.^28^

Finally, we demonstrated the robustness of results to possible unpublished null effect studies across long term, mid-term, and short term variability in blood pressure. Although results for long term variability may be considered conclusive, results for mid-term and short term variability are more susceptible to publication bias and may warrant further investigation.

### Limitations of this review

Included studies were primarily in older adults (mean age 48.5 to 77 years) and those at increased risk of cardiovascular disease (eg, due to hypertension) and conducted in European or East Asian populations. Hence the applicability of our findings to younger or healthier people and other ethnic groups is unknown. Studies in patients with a history of cerebrovascular events reported the largest hazard ratios, but significant associations remained after removal of these studies from analyses, and so findings remain applicable to people free from cerebrovascular disease. In studies in hypertensive patients,^14^ ^40^ ^69^ blood pressure variability could be confounded by entry criteria (regression to the mean)^71^ and treatment. However, such effects would diminish rather than exaggerate hazard ratios for variability, and so our overall conclusions are sound.

Lack of data from distinct cohorts prevented formal meta-analyses for many outcomes for to mid-term variability in blood pressure. A previous review was similarly limited by paucity of data,^26^ despite broader inclusion criteria. Our meta-analyses for short term variability in blood pressure were also dominated by two large studies. Despite these caveats, results supported an effect of shorter term variability on cardiovascular outcomes, and pooled hazard ratios were similar to those observed for long term variability. We were unable to determine if findings varied with timing and frequency of measurement.

In several analyses, there was significant heterogeneity between studies, potentially due to outlying studies in specific populations (eg, previous vascular disease) or to approximations necessary during data extraction, such as conversion from categorical (eg, from 10ths^10^ or thirds^49^) to continuous scale. However, not all converted hazard ratios were outliers,^50^ and we verified our conversion method in simulated data (not shown). Significant heterogeneity was reduced by removal of outlier studies, but this did not significantly alter the results.

In some cases, few studies contributed to main analyses, and the validity of these meta-analyses is debateable. Secondary analysis utilising data from all studies regardless of quality greatly increased the amount of available data but did not materially change results. Only three otherwise eligible studies failed to contribute any quantitative data, despite contact with authors.^44^ ^48^ ^52^

In general, there was poor reporting of study factors that may confound the relation between blood pressure variability and outcomes. Although studies were excluded from main analyses based on the three most important factors (prespecified), it was not feasible to do this for all factors. Further adjustment for confounders might be possible using individual patient data but was beyond the scope of this review. The importance of consideration and reporting of such confounding factors in future work on blood pressure variability (and variability in other biological measures) should be emphasised. Although our results indicate that these may be less important in the assessment of blood pressure variability, they may prove instrumental in other clinical areas.

### Clinical implications

The mechanism linking blood pressure variability to cardiovascular events is not well understood. Short term variability in blood pressure is affected by behavioural, emotional, and postural influences on cardiovascular physiology and cardiac rhythm.^72^ ^73^ Arterial stiffness contributes to both short term^74^ ^75^ and long term variability in blood pressure.^73^ ^76^ ^77^ Meanwhile, poor control of blood pressure resulting in changes to antihypertensive drugs also affects variability.^72^ Use of certain classes of antihypertensive drugs has also been linked with increased visit-to-visit variability^78^ and may not be entirely explained by adherence.^79^

The estimated standardised hazard ratio for the effect of long term variability in blood pressure on cardiovascular disease mortality was 1.18. For comparison, the effect of mean blood pressure on cardiovascular disease mortality reported in a previous meta-analysis^3^ corresponds to a standardised hazard ratio of approximately 1.7 (assuming a between person standard deviation of 15 mm Hg). Note that the latter standardised hazard ratio for mean blood pressure is not adjusted for variability, whereas the former (for blood pressure variability) is adjusted for mean blood pressure, showing the additional prognostic value of variability over and above the mean. This supports the results of recent work showing the improved discrimination of models including short term night-time variability in blood pressure^64^ or long term variability,^39^ over and above traditional risk factors.

How does blood pressure variability compare with other risk factors for cardiovascular disease? A recent review^80^ found that the adjusted standardised hazard ratio for increases in cholesterol on cardiovascular disease events varied between 1.16 and 1.29 in primary prevention groups, depending on the measure of cholesterol considered (eg, total cholesterol, triglycerides). Hence variability in blood pressure has similar prognostic value to cholesterol measures (standardised hazard ratio for long term variability on cardiovascular disease events=1.18).

Variability in blood pressure is not easily assessed clinically, and it is unclear if certain measures of variability should be preferred. Some measures could be calculated by hand (eg, average real variability), whereas others could be automatically calculated by electronic health records. This would enable doctors to account for both mean and variability in blood pressure concurrently when assessing cardiovascular risk. For example, assuming a standard deviation for variability (standard deviation) in systolic blood pressure of 5 mm Hg, an individual with variable blood pressure readings (139, 132, and 125 mm Hg, mean 132, SD 7) could be considered at 18% greater risk of cardiovascular disease events than a similar person with stable blood pressure (134, 130, and 132 mm Hg, mean 132, SD 2). This may be particularly important for patients with a highly variable but comparatively low mean blood pressure or for whom traditional cardiovascular risk estimates lie close to treatment thresholds. Further work is needed to determine the feasibility of obtaining such additional information, and the clinical impact on subsequent risk management.

### Conclusion

Long term variability in blood pressure measured in adults at clinic visits is associated with cardiovascular and mortality outcomes, over and above the effect of mean blood pressure. Mid-term (home monitoring) and short term (ambulatory monitoring) variability in blood pressure is also associated with all cause mortality, but the association with cardiovascular disease outcomes requires further investigation in novel cohorts.

**What is already known on this topic**

It is well established that patients with high blood pressure are at higher risk of future cardiovascular disease

Some studies have also suggested that patients with higher variability in blood pressure over time are at higher risk compared with patients with the same mean blood pressure level

It is not clear whether this risk depends on the method of measurement of variability, and few have correctly accounted for mean blood pressure or changes in treatment

**What this study adds**

Methodological errors are present in approximately half of prospective studies of blood pressure variability, but the association of long term (clinic) variability in blood pressure with future cardiovascular disease is found even in studies that avoid errors

Mid-term and short term variability in blood pressure measured at home or by ambulatory monitoring, respectively, has been little studied comparatively, but shows similar associations with outcomes

## Web extra

Extra material supplied by authors

Supplementary information: supplementary tables e1-e7 and figures e1-e18

## Notes

We thank Derek Shaw and David Yeomans, patient representatives, for their contribution to the design and content of the National Institute for Health Research (NIHR) programme grant from which this work arose. The protocol for this review has been published (www.crd.york.ac.uk/PROSPERO/ CRD42014015695).

Ethical approval: Not required.

Data sharing: Data extracted from the studies in this review are available on request from the corresponding author.

Transparency: The lead author (SS) affirms that the manuscript is an honest, accurate, and transparent account of the study being reported; that no important aspects of the study have been omitted; and that any discrepancies from the study as planned (and, if relevant, registered) have been explained
