# Supplementary material for: Blood pressure variability and cardiovascular disease: systematic review and meta-analysis
Source: BMJ. 2016 Aug 9;354:i4098. doi: 10.1136/bmj.i4098 (PMC4979357; doi:10.1136/bmj.i4098)
Supplement: Supplementary file 1 — Supplementary information: supplementary tables e1-e7 and figures e1-e18 [file stes029896.ww1_default.pdf]

Online supplement

Table e1: Search strategy

CINAHL (EBSCOHost)

S24	S22 OR S23 Limiters - Clinical Queries: Prognosis - Specificity
S23	S20 OR S21 Limiters - Clinical Queries: Prognosis - High Sensitivity
S22	S20 OR S21
S21	TI (((blood pressure or bp or sbp or dbp) N5 (variabilit* or variation*))) OR AB (((blood pressure or bp or sbp or dbp) N5 (variabilit* or variation*)))
S20	S3 AND S6 AND S19
S19	S7 OR S8 OR S9 OR S10 OR S11 OR S12 OR S13 OR S14 OR S15 OR S16 OR S17 OR S18
S18	TI within subject* OR AB within subject*
S17	TI ((dipping or dipper* or nondipping or nondipper* or non-dipping or non-dipper*)) OR AB ((dipping or dipper* or nondipping or nondipper* or non-dipping or non-dipper*))
S16	TI ((((daytime or day-time or diurnal) N5 (blood pressure or bp or sbp or sbp)) and ((night-time or nocturnal) N5 (blood pressure or bp or sbp or sbp)))) OR AB ((((daytime or day-time or diurnal) N5 (blood pressure or bp or sbp or sbp)) and ((night-time or nocturnal) N5 (blood pressure or bp or sbp or sbp))))
S15	TI (((daytime or day-time or diurnal) N5 (night-time or nocturnal))) OR AB (((daytime or day-time or diurnal) N5 (night-time or nocturnal)))
S14	TI repeat* measure* OR AB repeat* measure*
S13	TI "measure* to measure*" OR AB "measure* to measure*"
S12	TI (("day to day" or "day by day")) OR AB (("day to day" or "day by day"))
S11	TI ("between day" OR "within day") OR AB ("between day" OR "within day")
S10	TI "visit to visit" OR AB "visit to visit"
S9	TI (((between or within) N3 visit*)) OR AB (((between or within) N3 visit*))
S8	TI variation*
S7	TI (variability or variabilities) OR AB (variability or variabilities)
S6	S4 OR S5
S5	TI (blood pressure or bp or sbp or dbp) OR AB (blood pressure or bp or sbp or dbp)
S4	(MH "Blood Pressure") OR (MH "Blood Pressure Determination")
S3	S1 OR S2
S2	TI (hypertensive* or hypertension* or antihypertens* or anti-hypertens*) OR AB (hypertensive* or hypertension* or antihypertens* or anti-hypertens*)
S1	(MH "Hypertension+")

Embase (OvidSP)

1	*hypertension/
2	(hypertensive* or hypertension* or antihypertens* or anti-hypertens*).ti,ab.
3	1 or 2
4	*Blood Pressure/
5	exp *blood pressure measurement/
6	(blood pressure or bp or sbp or dbp).ti,ab.
7	4 or 5 or 6
8	(variability or variabilities).ti,ab.
9	variation?.ti.
10	((between or within) adj3 visit?).ti,ab.
11	"visit to visit".ti,ab.
12	((between or within) adj day?).ti,ab.
13	("day to day" or "day by day").ti,ab.
14	"measure* to measure*".ti,ab.
15	"reading? to reading?".ti,ab.
16	repeat* measure*.ti,ab.
17	((daytime or day-time or diurnal) adj5 (night-time or nocturnal)).ti,ab.
18	(((daytime or day-time or diurnal) adj5 (blood pressure or bp or sbp or sbp)) and ((night-time or nocturnal) adj5 (blood pressure or bp

	or sbp or sbp))).ti,ab.
19	(dipping or dipper? or nondipping or nondipper? or non-dipping or non-dipper?).ti,ab.
20	within subject?.ti,ab.
21	8 or 9 or 10 or 11 or 12 or 13 or 14 or 15 or 16 or 17 or 18 or 19 or 20
22	3 and 7 and 21
23	blood pressure variability/
24	3 and 23
25	((blood pressure or bp or sbp or dbp) adj5 (variabilit* or variation?)).ti,ab.
26	22 or 24 or 25
27	((exp animal/ or exp vertebrate/ or exp invertebrate/) not human/) or animal experiment/ or animal model/ or animal tissue/ or animal cell/ or nonhuman/
28	26 not 27
29	follow up.mp. or ep.fs. or prognos*.tw.
30	28 and 29
31	(prognos* or survival).tw.
32	28 and 31
33	30 or 32

Medline (OvidSP)

1	exp Hypertension/
2	(hypertensive* or hypertension* or antihypertens* or anti-hypertens*).ti,ab.
3	1 or 2
4	*Blood Pressure/
5	*Blood Pressure Determination/
6	(blood pressure or bp or sbp or dbp).ti,ab.
7	4 or 5 or 6
8	(variability or variabilities).ti,ab.
9	variation?.ti.
10	((between or within) adj3 visit?).ti,ab.
11	"visit to visit".ti,ab.
12	((between or within) adj day?).ti,ab.
13	("day to day" or "day by day").ti,ab.
14	"measure* to measure*".ti,ab.
15	"reading? to reading?".ti,ab.
16	repeat* measure*.ti,ab.
17	((daytime or day-time or diurnal) adj5 (night-time or nocturnal)).ti,ab.
18	(((daytime or day-time or diurnal) adj5 (blood pressure or bp or sbp or sbp)) and ((night-time or nocturnal) adj5 (blood pressure or bp or sbp or sbp))).ti,ab.
19	(dipping or dipper? or nondipping or nondipper? or non-dipping or non-dipper?).ti,ab.
20	within subject?.ti,ab.
21	8 or 9 or 10 or 11 or 12 or 13 or 14 or 15 or 16 or 17 or 18 or 19 or 20
22	3 and 7 and 21
23	((blood pressure or bp or sbp or dbp) adj5 (variabilit* or variation?)).ti,ab.
24	22 or 23
25	exp animal/ not human/
26	24 not 25
27	incidence.sh. or exp mortality/ or follow-up studies.sh. or prognos*.tw. or predict*.tw. or course*.tw.
28	26 and 27
29	(prognos* or first episode or cohort).tw.
30	26 and 29
31	28 or 30

Web of Science:

# 17	#16 AND #15
# 16	Topic=(prognos* OR cohort* OR incidence OR mortality OR "follow-up" OR predict OR course)
# 15	#14 AND #13
# 14	Topic=(hypertens* OR antihypertens* OR anti-hypertens*)
# 13	#12 OR #2 OR #1
# 12	#11 AND #3
# 11	#10 OR #9 OR #8 OR #5 OR #4
# 10	Topic=("within subject*")
# 9	Topic=(dipping or dipper* or nondipping or nondipper* or non-dipping or non-dipper*)
# 8	#7 AND #6
# 7	Topic=(nightime NEAR/5 ("blood pressure" or bp or sbp or dbp)) OR Topic=(nocturnal NEAR/5 ("blood pressure" or bp or sbp or dbp))
# 6	Topic=(daytime NEAR/5 ("blood pressure" or bp or sbp or dbp)) OR Topic=(day-time NEAR/5 ("blood pressure" or bp or sbp or dbp)) OR Topic=(diurnal NEAR/5 ("blood pressure" or bp or sbp or dbp))
# 5	Topic=((daytime NEAR/5 (night-time or nocturnal))) OR Topic=((day-time NEAR/5 (night-time or nocturnal))) OR Topic=((diurnal NEAR/5 (night-time or nocturnal)))
# 4	Topic=(between NEAR/3 visit*) OR Topic=(within NEAR/3 visit*) OR Topic=("between day*" OR "within day*") OR Topic=("day to day" OR "day by day") OR Topic=("measure to measure") OR Topic=("measurement to measurement") OR Topic=("repeat measur*" OR "repeated measur*") OR Topic=("visit to visit") OR Topic=("reading to reading" OR "readings to readings")
# 3	Topic=("blood pressure" OR bp OR sbp OR dbp)
# 2	Title=(variation* OR variability OR variabilities) AND Title=("blood pressure" OR bp OR sbp OR dbp)
# 1	Topic=("blood pressure" NEAR/5 (variability OR variabilities OR variation*)) OR Topic=(bp NEAR/5 (variability OR variabilities OR variation*)) OR Topic=(dbp NEAR/5 (variability OR variabilities OR variation*)) OR Topic=(sbp NEAR/5 (variability OR variabilities OR variation*))

Table e2: Extracted information

Patient characteristics

- Number of participants
- Age
- Proportion on anti-hypertensive medication
- Gender
- Source population

Study characteristics

- Type of monitoring
- Measurement device used*
- Person taking readings*
- Length of monitoring period
- Length of follow-up
- Authors overall conclusion
- Type of study (trial/ observational)
- Measurement arm*
- Cuff size used*
- Outcomes studied
- Variability measures studied

Statistical analysis

- Analysis strategy
- Variability measure
- Systolic or diastolic BP
- Reported hazard ratio/ 95% confidence interval
- Adjustment for equivalent mean BP*
- Regression to the mean considered*
- Medication change during measurement period limited/ adjusted for*
- Medication change during follow-up period limited/ adjusted for*
- Definition of a single measurement
- Outcome
- Units of reported hazard ratio
- Standard deviation of variability measure
- Diurnal/ Seasonal variation considered*

*considered as potential confounders of the effect of BP variability on outcomes.

Table e3: Standardized hazard ratios explanation

A standardized beta coefficient is calculated as the beta coefficient per unit of the standardized exposure (the exposure divided by its sample SD). For proportional hazards models, the beta-coefficient is the logarithm of the hazard ratio. For example, for Eguchi et al. (2012) reported that the standard deviation of clinic SBP standard deviation is 4.6 mm Hg and the univariate HR per 5 mm Hg of SBP SD is 1.158; hence the beta coefficient is 0.457per 5 mm Hg, the standardized beta coefficient is 0.421 per SD (of SD) and the standardized hazard ratio is 1.52 per SD. This has an interpretation as “hazard ratio per one SD of SBP SD”. Because our exposure measurements include SD of SBP, and the phrase “hazard ratio per one SD of SD” may not promote clarity, we use the term “standardized hazard ratio” rather than “hazard ratio per one SD”.

Table e4: Included study characteristics

Paper, Year (Country)	Study	Population	N	Frequency of measurement	Measure of Variability	Follow-up	Antihypertensive medication	Outcome Measure
**Short term BP variability measured through ambulatory BP monitoring (ABPM)**								
Bjorklund, 2004[1] (Sweden)	ULSAM (observational)	70 year old men (Uppsala Longitudinal Study of Adult Men). Mean age 71. 100% male.	872	Every 20 mins	SD	9.5 years (max, mean 6.6 +/- 2.1 years)	30.0%	CVD events
Eguchi, 2012[2] (Japan)	Observational	Asymptomatic patients aged 33-88 attending general internal medicine clinics at three institutes in Japan, for the evaluation and management of hypertension. Mean age 67. 38% male.	457	Every 30 mins	SD	66 months (mean)	55.6%	Hard and all CVD events
Gavish, 2009[3] (Israel)	Observational	Non pregnant, greater than 16 years old, good quality ABPM. Mean age 56. 45% male	3433	Every 20 mins (day) or 30 mins (night)	SD, CV, day/night SD ratio	7.6 years (mean, max 16 years)	59.0%	All-cause mortality
Gavish, 2015[4] (Israel)	Observational	Hypertensive patients included in the ambulatory BP (ABP) measurement service database. Mean age 57. 46% male. (analysis of subset of Gavish, 2009)	1246	Every 20 mins (day) or 30 mins (night)	SD, ratio of systolic and diastolic SD	5 years	61.0%	All-cause mortality
Hansen, 2010[5] (Worldwide)	IDACO (observational)	Multiple different populations in ABPM database. Mean age 53. 53% male. [IDACO]	8939	Every 30 mins	SD, ARV, mean of day and night SD	11.3 years (median)	19.6%	All-cause mortality, CVD mortality, CVD events, cardiac and coronary events
Kikuya, 2000[6] (Japan)	Ohasama (observational)	Japanese general population > 40 years (mean age 61.7, men: women=40:60)	1542	Every 30 mins	SD	8.5 years (mean)	30.9%	CVD mortality
Mancia, 2007[7] (Italy)	PAMELA (observational)	Randomly selected individuals in Milan aged 25-74 years. Mean age 51. 50% male.	2012	Every 20 mins	SD	148 months (max follow-up)	Not stated	All-cause mortality and CVD mortality

Paper, Year (Country)	Study	Population	N	Frequency of measurement	Measure of Variability	Follow-up	Antihypertensive medication	Outcome Measure
Mena, 2014[8] (Worldwide)	IDACO (observational)	Discovery data: - subset of IDACO, Copenhagen cohort subjects equally distributed among the 2 sexes and among 4 age wit complete ABPM readings groups (41, 51, 61, and 71 years). Test data: IDACO subjects 18+, at least 10 daytime readings, 5 night-time readings, and 48 readings over 24 hours and were not included in the discovery dataset. Mean age54. 54% male.	1254 (discovery data), 5353 (test data)	Every 15 to 30 minutes (day) and 30 to 60 mins (night)	ARV	10.2 years (median, test data)	21.3% (test data)	All-cause mortality, CVD mortality, CHD mortality, CVD events, CHD events, stroke events
Palatini, 2014[9] (Worldwide)	ABP-International (observational)	Ambulatory BP International Study: combination of 8 prospective studies of random samples of patients referred to hospital for hypertension. Untreated patients with entry office BP >140/90 mmHg. Mean age 51. 56% male.	7112	Every 10 to 30 mins (day) and 15 to 30 mins (night)	SD, CV	5.5 years (median)	No - untreated population	CVD events and CVD mortality
Pierdomenico, 2005[10] (Italy)	Abruzzo, Italy (observational)	Uncomplicated mild clinic hypertensives. Mean age 49. 54% male.	1088	Every 15 mins (day) and 30 mins (night)	SD	4.74 years (mean)	87% at follow-up	CVD events
Pierdomenico, 2006[11] (Italy)	Abruzzo, Italy (observational)	Hypertensive patients undergoing ABPM in Italy. Mean age 59. 47% male.	1472	Every 15 mins (day) and 30 mins (night)	SD	4.88 years (mean)	100.0%	CVD events
Pierdomenico, 2009[12] (Italy)	Abruzzo, Italy (observational)	Hypertensive patients age 40+ years who were referred for an outpatient evaluation for hypertension in Italy. Mean age 58. 49% male.	1280	Every 15 mins (day) and 30 mins (night)	SD, ARV	4.75 +/- 1.8 years (mean, range 0.2-7.5)	57.0%	CVD events
Pringle, 2003[13] (Europe)	Syst-Eur trial	Syst-Eur study. Elderly patients (60+) with isolated systolic hypertension. Median age 69.5. 39% male.	744	Every 30 mins	SD	4.4 years (median)	100% (384 on active treatment in trial)	Stroke events, CHD events and CVD mortality

Paper, Year (Country)	Study	Population	N	Frequency of measurement	Measure of Variability	Follow-up	Antihypertensive medication	Outcome Measure
Rothwell, 2010[14] (UK/ Scandinavia)	ASCOT-BPLA trial ABPM substudy (subset of stroke/TIA patients)	Patients with previous TIA or stroke	1905	Every 30 mins	SD, CV, VIM	5 years (median)	100.0%	Stroke events
Verdecchia, 2007[15] (Italy)	PIUMA (observational)	Initially untreated subjects with essential hypertension. Mean age = 51 yrs. Prevalence of women 47%	2649	Every 15 mins	SD	6 years (mean, max 16 years)	Untreated initially - subsequent antihypertensive use recorded	CVD events
**Long-term BP variability measured through clinic BP monitoring**								
Arashi, 2015[16] (Japan)	HIJ-CREATE trial	Participants of the Heart Institute of Japan Candesartan Randomised Trial for Evaluation in Coronary Artery Disease. Hospitalized patients with coronary artery disease and hypertension aged 20-80, June 2001 to April 2004. Mean age 48.5. 80% male.	1734	Every 6 months for a year, then every 12 months	SD, CV, VIM	4.2 years (median)	100% (trial of antihypertensives)	CVD events
Blacher, 2015[17] (France)	SU.FOL.OM3 trial	Participants from the SU.FOL.OM3 trial with experience of a coronary or cerebral ischemic acute event 1–12 months before inclusion. 45-80 years. Mean age 61. 80% male.	2157	Baseline then annually for 5 years	SD, CV	4.2±1.0 years (mean, max 5 years)	Yes - between 5% on alpha blockers to 69% on beta blockers.	CVD events
Carr, 2012[18] (UK)	MRC Elderly Trial	Hypertensive patients with a mean systolic BP at 160-209mmHg and diastolic BP < 115mmHg at entry	4396	Fortnightly basis for first month then monthly basis for 3 months, then 3 monthly	SR and RSV (Root successive variance = SR divided by BP at baseline)	5.8 years (mean)	100% (trial of antihypertensives)	Stroke events and CHD events

Paper, Year (Country)	Study	Population	N	Frequency of measurement	Measure of Variability	Follow-up	Antihypertensive medication	Outcome Measure
Eguchi, 2012[2] (Japan)	Observational	Asymptomatic patients aged 33-88 attending general internal medicine clinics at three institutes in Japan, for the evaluation and management of hypertension. Mean age 67. 38% male.	457	Every month	SD	66 months (mean)	55.6%	Hard and all CVD events
Gao, 2014[19] (USA)	Observational	US Primary care patients aged 60+ years approached for a depression screening study 1991-1993. Mean age 68. 31% male.	2906	At routine outpatient visits	RMSE (root mean squared error)	12.9 years (median)	89.7%	All-cause mortality, CHD events and stroke events
Hara, 2014[20] (Europe)	Syst-Eur trial	Patients aged 60+ with isolated systolic hypertension (SBP <160, DBP<95). Mean age 70. 33% male.	4695	Every 3 months	SD, CV, VIM, ARV, MMD (min-max difference)	2 years (median)	50% (randomised to active treatment)	CVD events and mortality
Hata, 2013[21] (Worldwide)	ADVANCE trial	Patients aged 55+ with type 2 diabetes and history of major macro- or micro- vascular disease. Mean age 66. 58% male.	8811	Months 3, 4 and 6 then every 6 months up to 24 months	SD, CV	2.4 years (median)	69% (at baseline but trial of antihypertensives)	All-cause mortality, CVD mortality, CVD events, stroke events and MI events
Hsieh, 2012[22] (Taiwan)	Observational	Patients with type 2 diabetes visiting the diabetic clinic in the Metabolism Division at Changhua Christian hospital Sept 2003-Apr 2005. Mean age 63.5. 43% male.	2161	Every 2-6 months	SD, CV	66.7 months (mean)	80.0%	All-cause and CVD mortality
Kawai, 2013[23] (Japan)	NOAH (observational)	Non-Invasive Atherosclerotic Evaluation in Hypertension study. Outpatients diagnosed with essential hypertension recruited between January 1998 and June 2004 at Osaka University Medical Hospital. Mena age 62. 53% male.	485	Every 1-2 months (6 visits total)	SD	7.59 years (mean)	47.3%	CVD events

Paper, Year (Country)	Study	Population	N	Frequency of measurement	Measure of Variability	Follow-up	Antihypertensive medication	Outcome Measure
Kostis, 2014[24] (USA)	SHEP (trial)	Systolic hypertension in the elderly program. Average age 72, 57% women and 15% black. USA.	4736	Baseline, months 1, 2 and 3, then every 3 months	VIM, rSSR (sum of squared deviations between average and trend predicted BP), VABS2 (variance of absolute difference between successive daily BP (VABS2)	17 years (max)	100.0%	CVD mortality
Lau, 2014a[25] (China)	Observational	Ischaemic stroke patients without atrial fibrillation, Hong Kong. Average age 71 years. 53% male.	632	Every 3-4 months	CV	76+/- 18 months (mean)	80.0%	All-cause and CVD mortality, nonfatal recurrent stroke and nonfatal acute coronary syndrome
Lau, 2014b[26] (China)	Observational	Patients with known history of coronary artery disease, ischaemic stroke or diabetes. Mean age 66. 68% male.	656	Every 3-4 months	SD	81 +/- 12 months (mean)	Yes - from 8% on alpha-blockers to 51% on beta blocker	CVD events
Mallamaci, 2013[27] (Italy)	Observational	Italians aged 18-75 with CKD stages 3 and 4, recruited in renal clinics from Oct 2005 to Nov 2007. Mean age 64. 59% male.	1618	Two visits per year for 3 years	SD, CV	37 months (median)	94.0%	All-cause mortality and CVD events
Mancia, 2012[28] (Europe)	ELSA trial	European Lacidipine Study on Atherosclerosis which randomized antihypertensive treatment for 4 years to mildly or moderately hypertensive patients at relatively low cardiovascular risk Mean age 56. 56% male.	1521	Every 6 months	SD, CV	4 years (max)	Yes (trial of antihypertensives)	CVD events

Paper, Year (Country)	Study	Population	N	Frequency of measurement	Measure of Variability	Follow-up	Antihypertensive medication	Outcome Measure
McMullan, 2013[29] (USA)	AASK (trial)	African American Study of Kidney Disease. African Americans with hypertensive nephropathy. Mean age 55 years, 62% men.	908	Months 4, 6, 8, 10 and 12	SD	52 months (median, max 75 months)	Yes (trial of antihypertensives)	All-cause mortality, CVD mortality, CVD events
Muntner, 2015[30] (USA)	ALLHAT (trial)	Participants from the Antihypertensive and Lipid Lowering Treatment to Prevent Heart Attack Trial. Mean age 66. 52% male.	25,814	At 6, 9, 12, 16, 20, 24 and 28 months	SD, ARV, VIM	2.7 to 2.9 years (mean: outcome dependent, 5.7 years max)	100% (trial of antihypertensives)	Fatal CHD or non-fatal MI, all-cause mortality, stroke and heart failure.
Poortvliet, 2012[31] (Ireland/ Netherlands)	PROSPER trial	Men and women aged 70-82 years in Scotland, Ireland and the Netherlands with either pre-existing vascular disease (coronary, cerebral, or peripheral) or at high risk due to smoking, hypertension or diabetes. Long-term follow-up: mean age 75, 48.5% male..	4819 (short-term follow-up) 1808 (long-term follow-up)	Every 3 months	SD, CV	2.3 years (mean, max 3 years, Scottish sub-group: max of 9.3 years (mean 7.1))	62.6% (short term follow up), 59.6%(long term follow up)	All-cause mortality, CVD mortality, stroke events, CHD events
Rakugi, 2015[32] (Japan)	COLM trial[33]	Participants in the Combination of OLMesartan and a calcium channel blocker (CCB) or a diuretic in Japanese elderly hypertensive patients (COLM) trial. Hypertensive patients ages 65–84 years with a history of cardiovascular disease and/or cardiovascular risk factors and hypertensive. Mean age 74. 51% male.	4876	At , 3 and 6 months, then every 6 months to at least 3 years	SD, VIM, ARV	3.3. years (median)	100% (trial of antihypertensives)	CVD events
Rossignol, 2015[34] (Worldwide)	HEAAL trial	Patients from the Heart failure Endpoint evaluation of Angiotensin II Antagonist Loasrtan (HEAAL) study - patients with HF classes II-IV, LVEF<40% or intolerance to ACEi. Mean age 64. 70% male.	3732	Three times in the first year then semi-annually	SD, CV, ARV	6.8 years	100% (trial of antihypertensives)	All-cause mortality or hospitalisation for worsening heart failure
Rothwell, 2010[14] (UK/ Scandinavia)	ASCOT-BPLA trial (subset of stroke/TIA patients)	Patients with previous TIA or stroke	2011	At baseline, 6 weeks, 3 months, 6 months, then every 6 months	SD, CV, VIM	5 years (median)	100% (trial of antihypertensives)	Stroke events

Paper, Year (Country)	Study	Population	N	Frequency of measurement	Measure of Variability	Follow-up	Antihypertensive medication	Outcome Measure
Rothwell, 2010[14] (Netherlands)	Dutch-TIA trial[35] (subset of stroke/TIA patients)	Patients with recent TIA or stroke	3150	Every 4 months for 2.6 years	SD, CV, VIM	2.6 years. (mean)	42.0%	Stroke events
Rothwell, 2010[14] (Europe)	ESPS-1 trial[36] (subset of stroke/TIA patients)	Patients with recent cerebrovascular event	2500	Every 3 months for 2 years	SD, CV, VIM	2 years (max)	Not stated	Stroke events
Rothwell, 2010[14] (UK)	UK-TIA trial[37] (subset of stroke/TIA patients)	Patients with history of TIA, mean age 60.3 years.	2006	Every 4 months for 2.6 years	SD, CV, VIM	3.3 years (median, max 6.67 years)	27.0%	Stroke events
Shimbo, 2012[38] (USA)	Women's health initiative (observational)	Post-menopausal patients enrolled in the women's health initiative.	58228	Annually. (Mean visits = 7.9)	SD	5.4 years (median)	Not stated	Stroke events
Suchy-Dicey, 2013[39] (USA)	Cardiovascular health study (observational)	Subjects who either used no antihypertensives during a 5 year baseline period or who used the same anti-hypertensive regimen during that period. Mean age 71 yrs. 95% white	2548	5 annual clinic visits	SD	9.9 years (mean)	38.4%	All-cause mortality, MI events and stroke events
Wei, 2013[40] (China)	PROBE trial	Hypertensive Chinese patients aged 70+. Mean age 77. 66% male.	724	Every 6 months	SD	4 years (mean)	100.0%	CVD events
Yu, 2014[41] (China)	Observational	Hypertensive patients with records in an electronic database for Shanghai, China, Jan 2005 - July 2011. Aged 18+, without history of stroke, and with at least 6 database BP readings on average no more than 6 months apart. Mean age 64. 46% male.	122,636	Every 6 months	SD, CV	48 months (mean, range 36-60 months)	Not stated	Stroke events
**Mid-term BP variability measured through home BP monitoring**								

Paper, Year (Country)	Study	Population	N	Frequency of measurement	Measure of Variability	Follow-up	Antihypertensive medication	Outcome Measure
Asayama, 2013[42] (Japan)	Ohasama (observational)	>35 years old, at home during working hours, not hospitalised, not incapacitated. Mean age 59. 39% male.	2421	Every morning and evening for 28 days	VIM, ARV, MMD (min-max difference)	12 years (median)	27.1%	All-cause mortality, CVD mortality and stroke events
Hashimoto, 2012[43] (Japan)	Ohasama (observational)	Japanese Men. Mean age 58.6 years. 100% men.	902	28 morning readings over 28 days	SD	13.1 years (median)	26.1%	All-cause mortality, CVD mortality, stroke mortality, MI mortality, stroke events
Johanssen, 2012[44] (Finland)	Health 2000 study (observational)	Finnish adults aged 45-74 years. Mean age 56. 44% male.	1866	7 consecutive days - 2 in the morning and 2 in the evening	SD, ARV	7.8 years (mean)	30.6%	CVD events and all-cause mortality
Kikuya, 2008[45] (Japan)	Ohasama (observational)	Japanese. Baseline age 35-96 years (mean age = 59.3 +/- 12.3 years). 60.5% women	2455	One reading every day for 4 weeks	SD	11.9 years (median)	72.6%	All-cause mortality, CVD mortality, Non-CVD mortality, stoke mortality, CHD mortality, MI mortality

Study references

1 Bjorkland K, Lind L, Berglund L, *et al.* Prognostic significance of 24-h ambulatory blood pressure characteristics for cardiovascular morbidity in a population of elderly men. *J Hypertens* 2004;**22**:1691–7.

2 Eguchi K, Hoshide S, Schwartz JE, *et al.* Visit-to-visit and ambulatory blood pressure variability as predictors of incident cardiovascular events in patients with hypertension. *Am J Hypertens* 2012;**25**:962–8.

3 Gavish B, Ben-Dov IZ, Kark JD, *et al.* The association of a simple blood pressure-independent parameter derived from ambulatory blood pressure variability with short-term mortality. *Hypertens Res* 2009;**32**:488–95. doi:10.1038/hr.2009.39

4 Gavish B, Bursztyn M. Blood pressure and heart period variability ratios derived from 24-h ambulatory measurements are predictors of all-cause mortality. *J Hypertens* 2015;**33**:491–8. doi:10.1097/HJH.0000000000000459

5 Hansen TW, Thijs L, Li Y, *et al.* Prognostic value of reading-to-reading blood pressure variability over 24 hours in 8938 subjects from 11 populations. *Hypertension* 2010;**55**:1049–57. doi:10.1161/HYPERTENSIONAHA.109.140798

6 Kikuya M, Hozawa a., Ohokubo T, *et al.* Prognostic Significance of Blood Pressure and Heart Rate Variabilities : The Ohasama Study. *Hypertension* 2000;**36**:901–6. doi:10.1161/01.HYP.36.5.901

7 Mancia G, Bombelli M, Facchetti R, *et al.* Long-term prognostic value of blood pressure variability in the general population: results of the Pressioni Arteriose Monitorate e Loro Associazioni Study. *Hypertension* 2007;**49**:1265–70. doi:10.1161/HYPERTENSIONAHA.107.088708

8 Mena LJ, Maestre GE, Hansen TW, *et al.* How many measurements are needed to estimate blood pressure variability without loss of prognostic information? *Am J Hypertens* 2014;**27**:46–55. doi:10.1093/ajh/hpt142

9 Palatini P, Reboldi G, Beilin LJ, *et al.* Added predictive value of night-time blood pressure variability for cardiovascular events and mortality: the Ambulatory Blood Pressure-International Study. *Hypertension* 2014;**64**:487–93. doi:10.1161/HYPERTENSIONAHA.114.03694

10 Pierdomenico SD, Lapenna D, Bucci A, *et al.* Blood pressure variability and prognosis in uncomplicated mild hypertension. *Am Heart J* 2005;**149**:934–8. doi:10.1016/j.ahj.2004.06.029

11 Pierdomenico SD, Lapenna D, Di Tommaso R, *et al.* Blood pressure variability and cardiovascular risk in treated hypertensive patients. *Am J Hypertens* 2006;**19**:991–7.

12 Pierdomenico SD, Di Nicola M, Esposito AL, *et al.* Prognostic value of different indices of blood pressure variability in hypertensive patients. *Am J Hypertens* 2009;**22**:842–7. doi:10.1038/ajh.2009.103

13 Pringle E, Phillips C, Thijs L, *et al.* Systolic blood pressure variability as a risk factor for stroke and cardiovascular mortality in the elderly hypertensive population. *J Hypertens* 2003;**21**:2251–7.

14 Rothwell PM, Howard SC, Dolan E, *et al.* Prognostic significance of visit-to-visit variability, maximum systolic blood pressure, and episodic hypertension. *Lancet* 2010;**375**:895–905. doi:10.1016/S0140-6736(10)60308-X

15 Verdecchia P, Angeli F, Gattobigio R, *et al.* Impact of blood pressure variability on cardiac and cerebrovascular complications in hypertension. *Am J Hypertens* 2007;**20**:154–61. doi:10.1016/j.amjhyper.2006.07.017

16 Arashi H, Ogawa H, Yamaguchi JI, *et al.* Impact of Visit-to-Visit Variability and Systolic Blood Pressure Control on Subsequent Outcomes in Hypertensive Patients With Coronary Artery Disease (from the HIJ-CREATE Substudy). *Am J Cardiol* 2015;**116**:236–42. doi:10.1016/j.amjcard.2015.04.011

17 Blacher J, Safar ME, Ly C, *et al.* Blood pressure variability: cardiovascular risk integrator or independent risk factor? *J Hum Hypertens* 2015;**29**:122–6. doi:10.1038/jhh.2014.44

18 Carr MJ, Bao Y, Pan J, *et al.* The predictive ability of blood pressure in elderly trial patients. *J Hypertens* 2012;**30**:1725–33.

19 Gao S, Hendrie HC, Wang C, *et al.* Redefined blood pressure variability measure and its association with mortality in elderly primary care patients. *Hypertension* 2014;**64**:45–52. doi:10.1161/HYPERTENSIONAHA.114.03576

20 Hara A, Thijs L, Asayama K, *et al.* Randomised double-blind comparison of placebo and active drugs for effects on risks associated with blood pressure variability in the Systolic Hypertension in Europe trial. *PLoS One* 2014;**9**:e103169. doi:10.1371/journal.pone.0103169

21 Hata J, Arima H, Rothwell PM, *et al.* Effects of visit-to-visit variability in systolic blood pressure on macrovascular and microvascular complications in patients with type 2 diabetes mellitus: The advance trial. *Circulation* 2013;**128**:1325–34. doi:10.1161/CIRCULATIONAHA.113.002717

22 Hsieh Y-T, Tu S-T, Cho T-J, *et al.* Visit-to-visit variability in blood pressure strongly predicts all-cause mortality in patients with type 2 diabetes: A 5·5-year prospective analysis. *Eur J Clin Invest* 2012;**42**:245–53.

23 Kawai T, Ohishi M, Ito N, *et al.* Alteration of vascular function is an important factor in the correlation between visit-to-visit blood pressure variability and cardiovascular disease. *J Hypertens* 2013;**31**:1387–95; discussion 1395. doi:10.1097/HJH.0b013e328360f796

24 Kostis JB, Sedjro JE, Cabrera J, *et al.* Visit-to-visit blood pressure variability and cardiovascular death in the Systolic Hypertension in the Elderly Program. *J Clin Hypertens (Greenwich)* 2014;**16**:34–40. doi:10.1111/jch.12230

25 Lau K-K, Wong Y-K, Teo K-C, *et al.* Long-term prognostic implications of visit-to-visit blood pressure variability in patients with ischemic stroke. *Am J Hypertens* 2014;**27**:1486–94. doi:10.1093/ajh/hpu070

26 Lau KK, Wong YK, Chan YH, *et al.* Visit-to-visit blood pressure variability as a prognostic marker in patients with cardiovascular and cerebrovascular diseases - Relationships and comparisons with vascular markers of atherosclerosis. *Atherosclerosis* 2014;**235**:230–5. doi:10.1016/j.atherosclerosis.2014.04.015

27 Mallamaci F, Minutolo R, Leonardis D, *et al.* Long-term visit-to-visit office blood pressure variability increases the risk of adverse cardiovascular outcomes in patients with chronic kidney disease. *Kidney Int* 2013;**84**:381–9. doi:10.1038/ki.2013.132

28 Mancia G, Facchetti R, Parati G, *et al.* Visit-to-visit blood pressure variability in the European Lacidipine Study on Atherosclerosis: Methodological aspects and effects of antihypertensive treatment. *J Hypertens* 2012;**30**:1241–51.

29 McMullan CJ, Bakris GL, Phillips R a, *et al.* Association of BP variability with mortality among African Americans with CKD. *Clin J Am Soc Nephrol* 2013;**8**:731–8. doi:10.2215/CJN.10131012

30 Muntner P, Whittle J, Lynch AI, *et al.* Visit-to-Visit Variability of Blood Pressure and Coronary Heart Disease, Stroke, Heart Failure, and Mortality. *Ann Intern Med* 2015;**163**:329. doi:10.7326/M14-2803

31 Poortvliet RKE, Ford I, Lloyd SM, *et al.* Blood Pressure Variability and Cardiovascular Risk in the PROspective Study of Pravastatin in the Elderly at Risk (PROSPER). *PLoS One* 2012;**7**.

32 Rakugi H, Ogihara T, Saruta T, *et al.* Preferable effects of olmesartan/calcium channel blocker to olmesartan/diuretic on blood pressure variability in very elderly hypertension. *J Hypertens* 2015;**33**:2165–72. doi:10.1097/HJH.0000000000000668

33 Ogihara T, Saruta T, Rakugi H, *et al.* Rationale, study design and implementation of the COLM study: the combination of OLMesartan and calcium channel blocker or diuretic in high-risk elderly hypertensive patients. *Hypertens Res* 2009;**32**:163–7. doi:10.1038/hr.2008.31

34 Rossignol P, Girerd N, Gregory D, *et al.* Increased visit-to-visit blood pressure variability is associated with worse cardiovascular outcomes in low ejection fraction heart failure patients: Insights from the HEAAL study. *Int J Cardiol* 2015;**187**:183–9. doi:10.1016/j.ijcard.2015.03.169

35 Dutch TIA Trial Study Group. A comparison of two doses of aspirin (30 mg vs. 283 mg a day) in patients after a transient ischemic attack or minor ischemic stroke. The Dutch TIA Trial Study Group. *N Engl J Med* 1991;**325**:1261–6. doi:10.1056/NEJM199110313251801

36 THE ESPS Group. THE EUROPEAN STROKE PREVENTION STUDY (ESPS). *Lancet* 1987;**330**:1351–4. doi:10.1016/S0140-6736(87)91254-2

37 Farrell B, Godwin J, Richards S, *et al.* The United Kingdom transient ischaemic attack (UK-TIA) aspirin trial: final results. *J Neurol Neurosurg Psychiatry* 1991;**54**:1044–54.

38 Shimbo D, Newman JD, Aragaki AK, *et al.* Association between annual visit-to-visit blood pressure variability and stroke in postmenopausal women: Data from the Women’s health initiative. *Hypertension* 2012;**60**:625–30.

39 Suchy-Dicey AM, Wallace ER, Mitchell SVE, *et al.* Blood pressure variability and the risk of all-cause mortality, incident myocardial infarction, and incident stroke in the cardiovascular health study. *Am J Hypertens* 2013;**26**:1210–7. doi:10.1093/ajh/hpt092

40 Wei Y, Jin Z, Shen G, *et al.* Effects of intensive antihypertensive treatment on Chinese hypertensive patients older than 70 years. *J Clin Hypertens (Greenwich)* 2013;**15**:420–7. doi:10.1111/jch.12094

41 Yu J, Kong Q, Schoenhagen P, *et al.* The prognostic value of long-term visit-to-visit blood pressure variability on stroke in real-world practice: A dynamic cohort study in a large representative sample of Chinese hypertensive population. *Int J Cardiol* 2014;**177**:995–1000. doi:10.1016/j.ijcard.2014.09.149

42 Asayama K, Kikuya M, Schutte R, *et al.* Home blood pressure variability as cardiovascular risk factor in the population of ohasama. *Hypertension* 2013;**61**:61–9. doi:10.1161/HYPERTENSIONAHA.111.00138

43 Hashimoto T, Kikuya M, Ohkubo T, *et al.* Home blood pressure level, blood pressure variability, smoking, and stroke risk in Japanese men: the Ohasama study. *Am J Hypertens* 2012;**25**:883–91.

44 Johansson JK, Niiranen TJ, Puukka PJ, *et al.* Prognostic value of the variability in home-measured blood pressure and heart rate: the Finn-Home Study. *Hypertension* 2012;**59**:212–8. doi:10.1161/HYPERTENSIONAHA.111.178657

45 Kikuya M, Ohkubo T, Metoki H, *et al.* Day-by-day variability of blood pressure and heart rate at home as a novel predictor of prognosis: the Ohasama study. *Hypertension* 2008;**52**:1045–50.

Table e5: Study and analysis characteristics that may confound the relationship between blood pressure variability and outcomes

Paper, Year	Study design characteristics				Potential confounders							
	Appropriate cuff size used	Consistent reading arm	Consistent device used	Same person taking readings	Appropriate adjustment for mean BP	Regression to mean considered (if relevant)	Diurnal/ seasonal variation considered	Medication change during measurement period limited	Medication change during follow-up limited	Measurement before follow-up	Definition of a single measurement given	Main analysis (yes or reason for exclusion)
**Short-term BP variability measured through ambulatory BP monitoring (ABPM)**												
Bjorklund, 2004	Unclear	Yes	Mercury sphyg	Yes (ABPM)	No	Not relevant	Yes	No but ABPM	No	Yes (ABPM)	Yes	No adjustment for mean BP
Eguchi, 2012: ABPM analyses	Unclear	Unclear	Yes	Yes (ABPM)	No –adjusted for clinic mean	Not relevant	No	No but ABPM	No	Yes (ABPM)	Yes	ABPM analyses adjusted for clinic mean
Gavish, 2009	Yes	Yes	No	Yes (ABPM)	No -adjusted for mean arterial pressure	No	No	No but ABPM	No	Yes (ABPM)	Yes	Incorrect adjustment for mean BP
Gavish, 2015	Yes	Yes	No	Yes	Yes	Not relevant	No	No but ABPM	No	Yes (ABPM)	Yes	Yes
Hansen, 2010	Unclear	Unclear	Unclear	Yes (ABPM)	Yes	Not relevant	Yes	No but ABPM	No	Yes (ABPM)	Yes	Yes
Kikuya, 2000	Unclear	Unclear	Yes	Yes (ABPM)	No -adjusted for 24-hour BP in day/night analysis	Not relevant	Yes	No but ABPM	No	Yes (ABPM)	Yes	Adjusted for 24-hour BP in day/night analysis
Mancia, 2007	Unclear	Unclear	Yes	Yes (ABPM)	No - adjusted for 24-hour mean in day/ night analysis	Not relevant	Yes	No but ABPM	No	Yes (ABPM)	Yes	Adjusted for 24-hour mean in day/ night analysis
Mena, 2014	Yes	Unclear	Unclear	Yes (ABPM)	Yes	Not relevant	No	No but ABPM	No	Yes	Yes	Yes
Palatini, 2014	Unclear	Unclear	Unclear	Yes (ABPM)	Yes	Not relevant	Yes	Yes - untreated patients	No	Yes (ABPM)	Yes	Yes

Paper, Year	Study design characteristics				Potential confounders							
	Appropriate cuff size used	Consistent reading arm	Consistent device used	Same person taking readings	Appropriate adjustment for mean BP	Regression to mean considered (if relevant)	Diurnal/ seasonal variation considered	Medication change during measurement period limited	Medication change during follow-up limited	Measurement before follow-up	Definition of a single measurement given	Main analysis (yes or reason for exclusion)
Pierdomenico, 2005	Unclear	Unclear	Yes	Yes (ABPM)	Yes	Not relevant	No	No but ABPM	No	Yes (ABPM)	Yes	No extractable data
Pierdomenico, 2006	Yes	Unclear	Yes	Yes (ABPM)	Yes	Not relevant	Yes	No but ABPM	No	Yes (ABPM)	Yes	Yes
Pierdomenico, 2009	Yes	Unclear	Yes	Yes (ABPM)	Yes	Not relevant	Yes	No but ABPM	No	Yes (ABPM)	Yes	Yes
Pringle, 2003	Yes	Yes	No	Yes (ABPM)	Yes	Not relevant	Yes	No but ABPM	No	Yes (ABPM)	Yes	Yes
Rothwell, 2010: ASCOT-BPLA ABPM substudy	Unclear	Unclear	Yes	Yes (ABPM)	Unclear	No	Yes	Yes (ABPM)	No	Yes (ABPM)	Yes	Adjustment for mean unclear
Verdecchia, 2007	Unclear	Unclear	No	Yes (ABPM)	Yes	Not relevant	Yes	No but ABPM	No	Yes (ABPM)	Yes	Yes
**Long-term BP variability measured through clinic BP monitoring**												
Arashi, 2015	Unclear	Unclear	Mercury sphyg	Unclear	No - diastolic analysis adjusted for systolic mean	Yes	No	No	No	No	Unclear	Follow-up and measurement confounded
Blacher, 2015	Unclear	Unclear	Yes	Unclear	Yes	Not relevant	No	No	No	Unclear	Yes	Follow-up and measurement confounded
Carr, 2012	Unclear	Unclear	Yes	No	Yes	Yes	Yes	No	No	No	Yes	Treatment adherence low
Eguchi, 2012: clinic analyses	Unclear	Unclear	Mercury sphyg	Unclear	Yes	Not relevant	No	No	No	Unclear	Yes	Measurement and follow-up confounded
Gao, 2014	Unclear	Unclear	Unclear	Unclear	Yes	Not relevant	No	No	No	No	Unclear	Follow-up and measurement confounded

Paper, Year	Study design characteristics				Potential confounders							
	Appropriate cuff size used	Consistent reading arm	Consistent device used	Same person taking readings	Appropriate adjustment for mean BP	Regression to mean considered (if relevant)	Diurnal/ seasonal variation considered	Medication change during measurement period limited	Medication change during follow-up limited	Measurement before follow-up	Definition of a single measurement given	Main analysis (yes or reason for exclusion)
Hara, 2014	Yes	Yes	Mercury sphyg	Unclear	Yes	Yes	No	No but adjusted for in secondary analysis	Yes	No	Yes	Follow-up and measurement confounded
Hata, 2013	Unclear	Unclear	Yes	Unclear	Yes	Not relevant	No	No but results similar in those who did not change	No	Yes	Yes	Yes
Hsieh, 2012	Yes	Unclear	Yes	Unclear	Yes	Not relevant	No	No	No	Unclear	Yes	No extractable data
Kawai, 2013	Unclear	Unclear	No	Unclear	Yes	Not relevant	No	Yes	No	Unclear	Yes	Follow-up and measurement confounded
Kostis, 2014	Unclear	Unclear	Yes	Unclear	Yes	No	No	No but adherence high, results in cross-over patients similar	No	No	Yes	Yes
Lau, 2014a	Unclear	Unclear	Yes	Unclear	Yes	Not relevant	No	No but adjusted for medication use	No	Unclear	Yes	Follow-up and measurement confounded
Lau, 2014b	Unclear	Unclear	Yes	Unclear	Yes	Not relevant	No	No - only adjusted for baseline medication	No	Unclear	Yes	Follow-up and measurement confounded
Mallamaci, 2013	Yes	Yes	Mercury sphyg	No	Yes	Not relevant	No	No	No	Unclear	Yes	Follow-up and measurement confounded
Mancia, 2012	Unclear	Unclear	Mercury sphyg	Unclear	Yes	No	No	Yes	Yes	No	Yes	No extractable data
McMullan, 2013	Unclear	Unclear	Yes	Unclear	Yes	Not relevant	No	No (adherence low)	No	Yes	Yes	Medication adherence low
Muntner, 2015	Unclear	Unclear	Unclear	Unclear	Yes	Yes	No	No but adjusted for medication use	No	Yes	Yes	Yes
Poortvliet, 2012	Unclear	Unclear	Yes	Unclear	Yes	Not relevant	No	No but trial of statins	No	Yes	Yes	Yes

Paper, Year	Study design characteristics				Potential confounders							
	**Appropriate cuff size used**	**Consistent reading arm**	**Consistent device used**	**Same person taking readings**	**Appropriate adjustment for mean BP**	**Regression to mean considered (if relevant)**	**Diurnal/ seasonal variation considered**	**Medication change during measurement period limited**	**Medication change during follow-up limited**	**Measurement before follow-up**	**Definition of a single measurement given**	**Main analysis (yes or reason for exclusion)**
Rakugi, 2015	Unclear	Unclear	Unclear	Unclear	Yes	No	No	Unclear (trial of antihypertensives, adherence unclear)	No	No	Unclear	Measurement and follow-up confounded
Rossignol, 2015	Unclear	Yes	No	Unclear	No	Not relevant	No	Unclear (trial of antihypertensives, adherence unclear)	Unclear	No	Unclear	No extractable data for review outcomes
Rothwell, 2010: ASCOT-BPLA	Unclear	Unclear	Yes	Unclear	Yes	No	No	No	No	Unclear	Yes	Follow-up and measurement confounded
Rothwell, 2010: Dutch TIA	Unclear	Unclear	Mercury sphyg	Unclear	Yes	Not relevant	No	No	No	Unclear	Yes	Follow-up and measurement confounded
Rothwell, 2010: ESPS-1	Unclear	Yes	Mercury sphyg	Unclear	Yes	Not relevant	No	Analysis of placebo group only	Yes	Unclear	Yes	Follow-up and measurement confounded
Rothwell, 2010: UK-TIA	Unclear	Unclear	Mercury sphyg	Unclear	Yes	Not relevant	No	RCT of aspirin only	No	Yes	Yes	Yes
Shimbo, 2012	Yes	Yes	Mercury sphyg	Unclear	Yes	Not relevant	No	No but medication adjusted for in analysis	Yes	Yes	Yes	Yes
Suchy-Dicey, 2013	Unclear	Yes	Mercury sphyg	Unclear	Yes	Not relevant	No	Yes (users of changing medication excluded)	No	Yes	Yes	Yes
Wei, 2013	Unclear	Yes	Manual sphyg	Unclear	Yes	Not relevant	No	No	No	Unclear	Yes	Follow-up and measurement confounded
Yu, 2014	Yes	Yes	Mercury sphyg	Unclear	Yes	No	No	No	No	No	Yes	Follow-up and measurement confounded
**Mid-term BP variability measured through home BP monitoring**												

Paper, Year	Study design characteristics				Potential confounders							
	**Appropriate cuff size used**	**Consistent reading arm**	**Consistent device used**	**Same person taking readings**	**Appropriate adjustment for mean BP**	**Regression to mean considered (if relevant)**	**Diurnal/ seasonal variation considered**	**Medication change during measurement period limited**	**Medication change during follow-up limited**	**Measurement before follow-up**	**Definition of a single measurement given**	**Main analysis (yes or reason for exclusion)**
Asayama, 2013	Unclear	Unclear	Yes	Yes (home)	Yes	Not relevant	Yes	Yes	No	Yes (home)	Yes	Yes
Hashimoto, 2012	Unclear	Unclear	Yes	Yes (home)	Yes	Not relevant	No	No but home	No	Yes (home)	Yes	Yes
Johanssen, 2012	Unclear	Unclear	Yes	Yes (home)	Yes	Not relevant	Yes	No but home	No	Yes (home)	Yes	Yes
Kikuya, 2008	Unclear	Unclear	Yes	Yes (home)	Yes	No	No	No but adjusted for medication use	No	Yes (home)	Yes	Yes

Table e6: Risk of bias assessment

Paper, Year	Study	Study Participation	Study Attrition	Prognostic Factor Measurement	Outcome Measurement	Study Confounding	Statistical Analysis and Reporting
**Short-term BP variability measured through ABPM**							
Bjorklund, 2004	ULSAM (observational)	low	moderate	low	low	moderate	low
Eguchi, 2012	Observational (ABPM)	moderate	low	low	low	moderate	low
Gavish, 2009	Observational	moderate	low	moderate	low	moderate	low
Gavish, 2015	Observational	moderate	low	moderate	low	low	low
Hansen, 2010	IDACO (observational)	moderate	low	low	low	low	low
Kikuya, 2000	Ohasama (observational)	moderate	low	low	low	moderate	low
Mancia, 2007	PAMELA (observational)	moderate	low	low	low	moderate	low
Mena, 2014	IDACO (observational)	moderate	low	low	low	low	low
Palatini, 2014	ABP-International (observational)	moderate	low	low	low	low	low
Pierdomenico, 2005	Abruzzo, Italy (observational)	moderate	moderate	low	moderate	low	high
Pierdomenico, 2006	Abruzzo, Italy (observational)	moderate	moderate	low	moderate	low	low
Pierdomenico, 2009	Abruzzo, Italy (observational)	moderate	moderate	low	moderate	low	low
Pringle, 2003	Syst-Eur trial	moderate	low	moderate	low	low	low
Rothwell, 2010	ASCOT-BPLA trial ABPM substudy (subset of stroke/TIA patients)	low	low	low	low	moderate	low
Verdecchia, 2007	PIUMA (observational)	moderate	low	low	moderate	low	moderate
**Long-term BP variability measured through clinic BP monitoring**							
Arashi, 2015	HIJ-CREATE trial	moderate	moderate	moderate	high	moderate	low
Blacher, 2015	SU.FOL.OM3 trial	low	low	low	high	low	high
Carr, 2012	MRC Elderly Trial	moderate	moderate	moderate	moderate	low	low
Eguchi, 2012	Observational (clinic)	moderate	low	low	high	low	low
Gao, 2014	Observational	low	low	moderate	high	high	low
Hara, 2014	Syst-Eur trial	moderate	low	low	high	low	low
Hata, 2013	ADVANCE trial	low	low	low	low	low	low
Hsieh, 2012	Observational	low	low	low	high	low	low
Kawai, 2013	NOAH (observational)	low	moderate	moderate	moderate	low	low
Kostis, 2014	SHEP (trial)	moderate	low	moderate	low	low	low
Lau, 2014a	Observational	low	high	low	high	low	low
Lau, 2014b	Observational	low	low	low	high	low	low
Mallamaci, 2013	Observational	moderate	low	moderate	high	low	low

Paper, Year	Study	Study Participation	Study Attrition	Prognostic Factor Measurement	Outcome Measurement	Study Confounding	Statistical Analysis and Reporting
Mancia, 2012	ELSA trial	moderate	low	low	high	low	moderate
McMullan, 2013	AASK (trial)	moderate	low	moderate	moderate	low	low
Muntner, 2015	ALLHAT (trial)	low	low	low	low	low	low
Poortvliet, 2012	PROSPER trial	low	low	low	low	low	low
Rakugi, 2015	COLM trial[33]	low	low	high	high	low	moderate
Rossignol, 2015	HEAAL trial	moderate	low	high	high	high	moderate
Rothwell, 2010	ASCOT-BPLA trial (subset of stroke/TIA patients)	moderate	low	low	high	low	low
Rothwell, 2010	Dutch-TIA trial[35] (subset of stroke/TIA patients)	low	low	moderate	high	low	low
Rothwell, 2010	ESPS-1 trial[36] (subset of stroke/TIA patients)	low	low	moderate	high	low	low
Rothwell, 2010	UK-TIA trial[37] (subset of stroke/TIA patients)	low	low	moderate	low	low	low
Shimbo, 2012	Women's health initiative (observational)	low	moderate	moderate	low	low	low
Suchy-Dicey, 2013	Cardiovascular health study (observational)	moderate	low	moderate	low	low	low
Wei, 2013	PROBE trial	low	moderate	moderate	high	moderate	low
Yu, 2014	Observational	low	moderate	low	high	low	low
**Mid-term BP variability measured through home BP monitoring**							
Asayama, 2013	Ohasama (observational)	moderate	low	low	low	low	low
Hashimoto, 2012	Ohasama (observational)	moderate	low	low	low	low	low
Johanssen, 2012	Health 2000 study (observational)	low	low	low	low	low	low
Kikuya, 2008	Ohasama (observational)	moderate	moderate	low	low	low	low

Supplementary data – long term variability in clinic BP

Figure e1

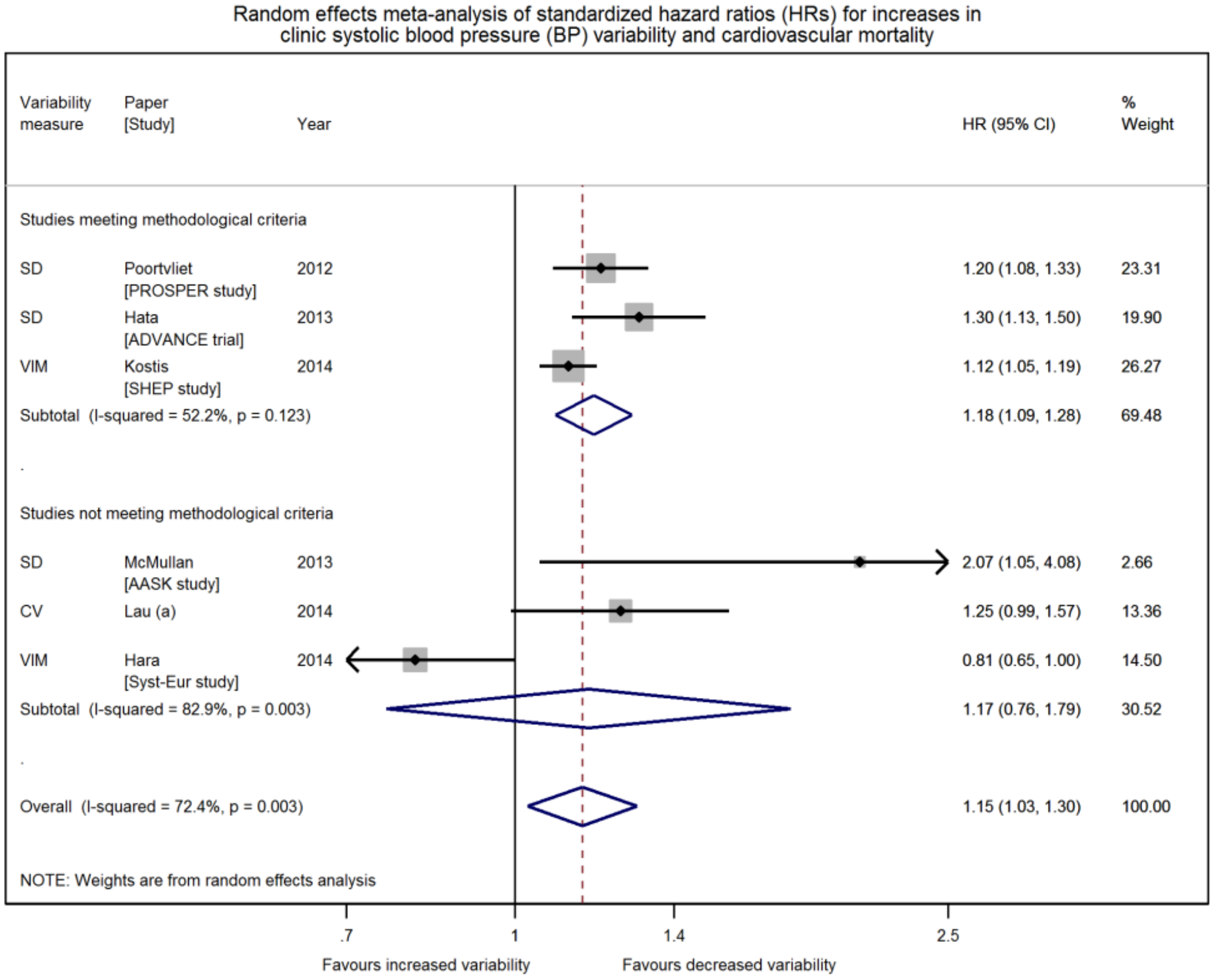

SD: Standard deviation, VIM: Variation independent of mean, CV: Coefficient of variation

Figure e2

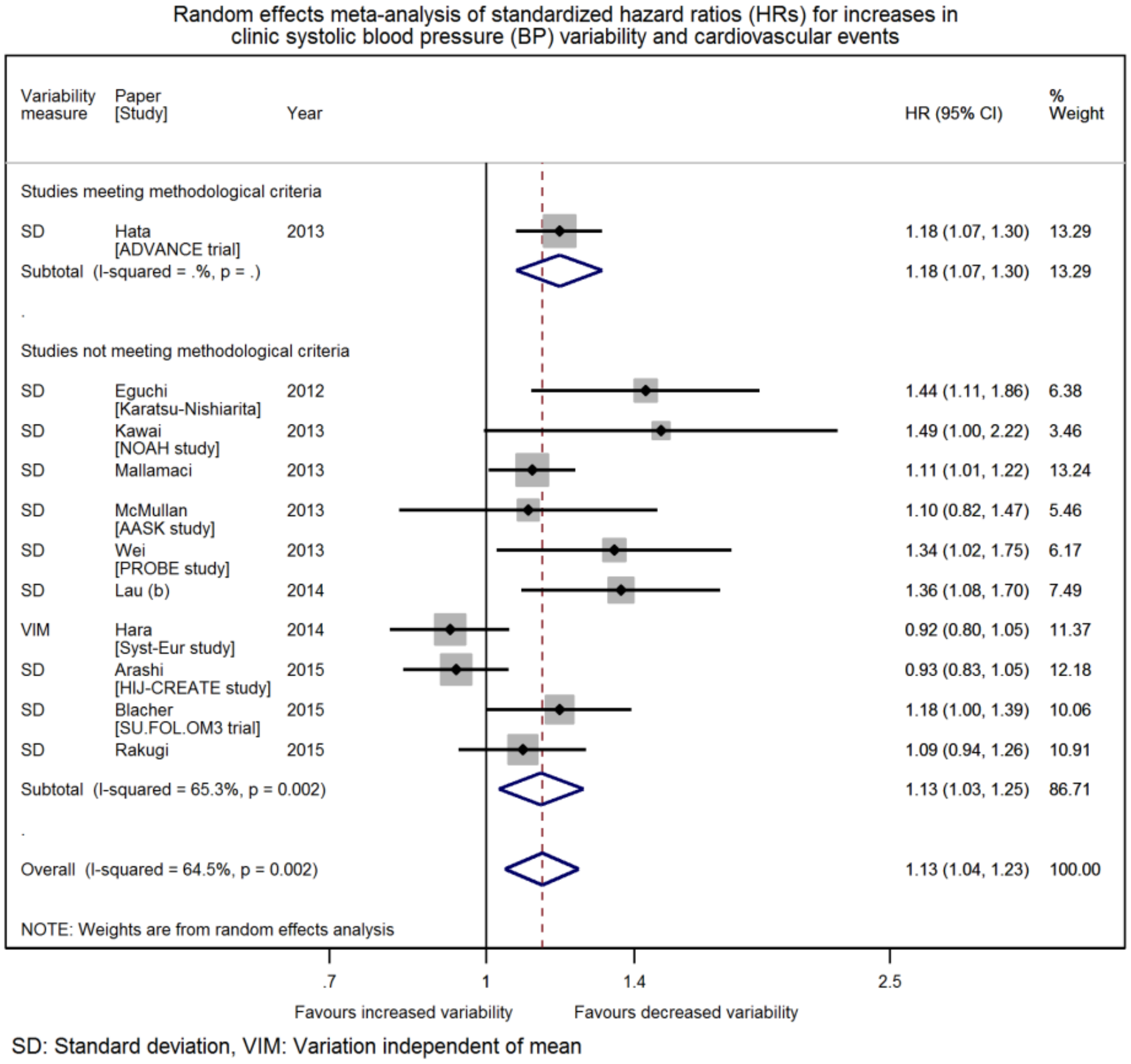
Random effects meta-analysis of standardized hazard ratios (HRs) for increases in clinic systolic blood pressure (BP) variability and cardiovascular events
Variability measure
Paper [Study]
Year
HR (95% CI)
% Weight
Studies meeting methodological criteria
SD Hata [ADVANCE trial] 2013 1.18 (1.07, 1.30) 13.29
Subtotal (I-squared = .%, p = .) 1.18 (1.07, 1.30) 13.29
.
Studies not meeting methodological criteria
SD Eguchi [Karatsu-Nishiarita] 2012 1.44 (1.11, 1.86) 6.38
SD Kawai [NOAH study] 2013 1.49 (1.00, 2.22) 3.46
SD Mallamaci 2013 1.11 (1.01, 1.22) 13.24
SD McMullan [AASK study] 2013 1.10 (0.82, 1.47) 5.46
SD Wei [PROBE study] 2013 1.34 (1.02, 1.75) 6.17
SD Lau (b) 2014 1.36 (1.08, 1.70) 7.49
VIM Hara [Syst-Eur study] 2014 0.92 (0.80, 1.05) 11.37
SD Arashi [HIJ-CREATE study] 2015 0.93 (0.83, 1.05) 12.18
SD Blacher [SU.FOL.OM3 trial] 2015 1.18 (1.00, 1.39) 10.06
SD Rakugi 2015 1.09 (0.94, 1.26) 10.91
Subtotal (I-squared = 65.3%, p = 0.002) 1.13 (1.03, 1.25) 86.71
.
Overall (I-squared = 64.5%, p = 0.002) 1.13 (1.04, 1.23) 100.00
NOTE: Weights are from random effects analysis
.7
1
1.4
2.5
Favours increased variability
Favours decreased variability
SD: Standard deviation, VIM: Variation independent of mean

Figure e3

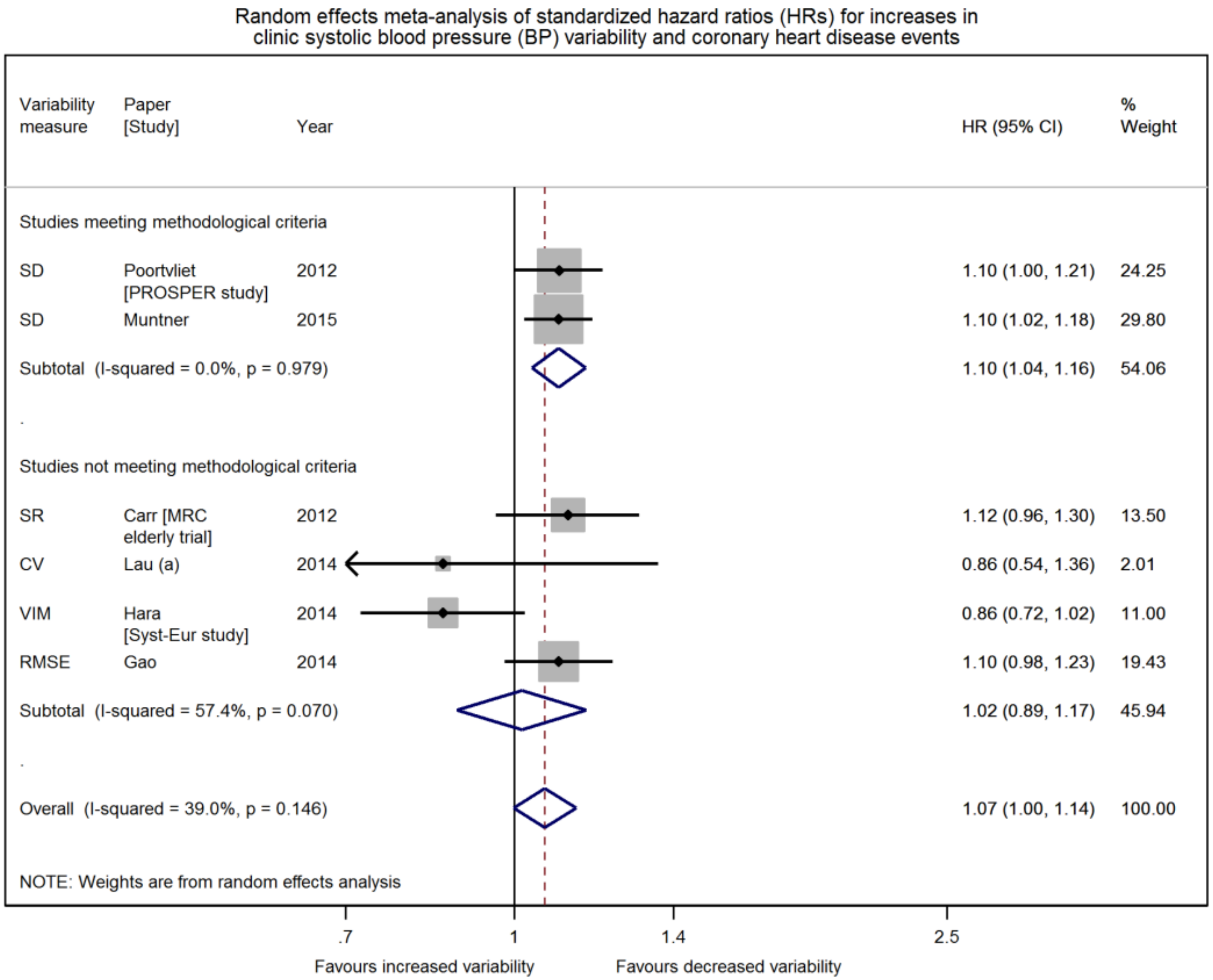

SD: Standard deviation, SR: Standardized residual, CV: Coefficient of variation, VIM: Variation independent of mean, RMSE: Root mean squared error

Figure e4

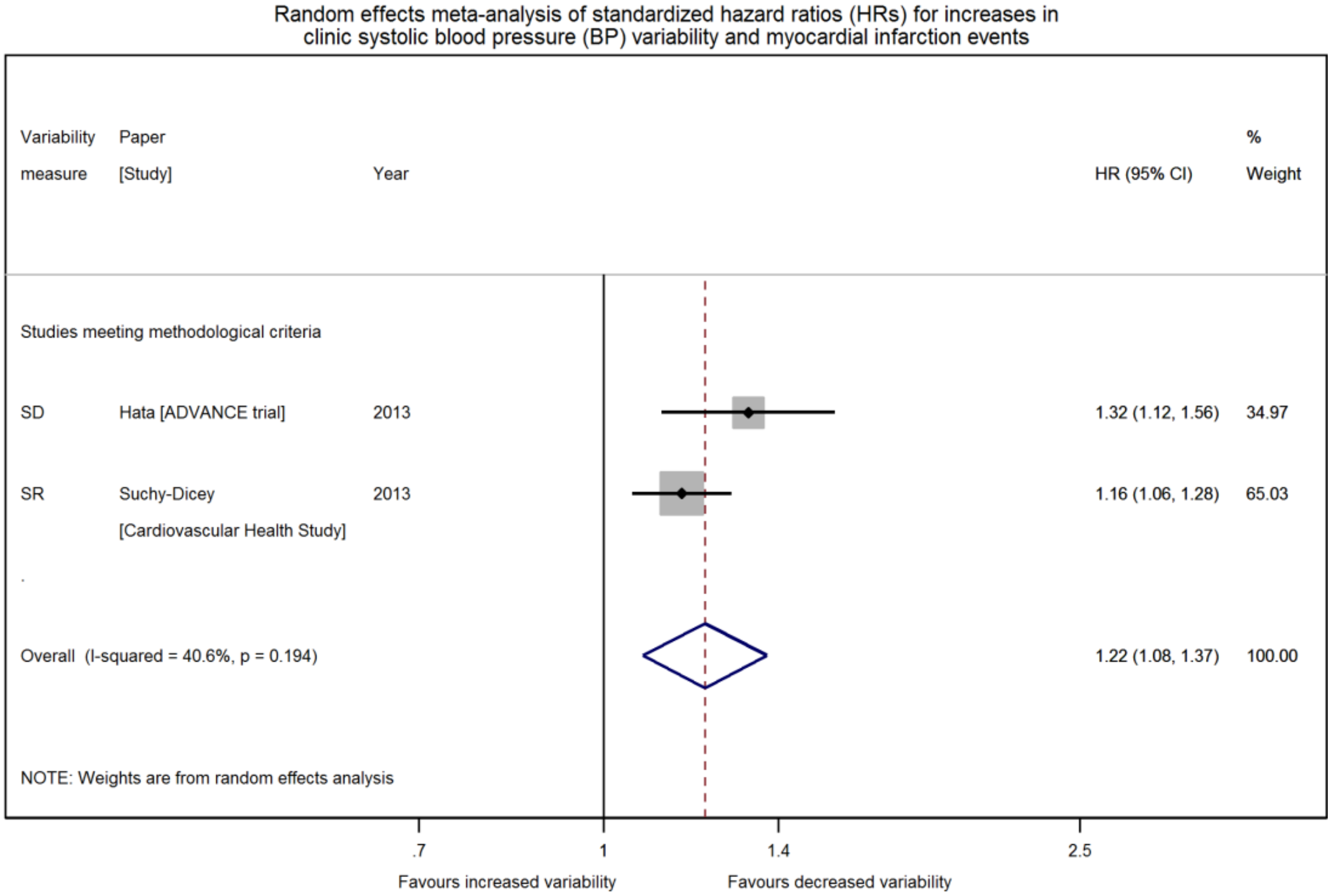

SD: Standard deviation, SR: Standardised residual

Supplementary data – mid-term variability in home BP

Table e7: Hazard ratios (HRs) for cardiovascular and mortality outcomes per standard deviation increase in home systolic blood pressure (BP) variability

	**Morning measurements**			**Evening measurements**			**Morning and evening measurements**		
**Outcome**	**Variability measure**	**Paper [Study], year**	**HR (95% CI)**	**Variability measure**	**Paper [Study], year**	**HR (95% CI)**	**Variability measure**	**Paper [Study], year**	**HR (95% CI)**
**CVD mortality**	VIM	Asayama [Ohasama], 2013	1.26 (1.07, 1.49)	VIM	Asayama [Ohasama], 2013	1.23 (1.05, 1.45)	SD	Kikuya [Ohasama], 2008	1.16 (0.99, 1.36)
**CHD mortality**	SD	Hashimoto [Ohasama], 2012	0.84 (0.59, 1.19)	SD	Kikuya [Ohasama], 2008	0.99 (0.79, 1.25)	SD	Kikuya [Ohasama], 2008	1.02 (0.81, 1.29)
**Stroke mortality**	SD	Hashimoto [Ohasama], 2012	1.47 (1.11, 1.95)	SD	Kikuya [Ohasama], 2008	1.38 (1.12, 1.70)	SD	Kikuya [Ohasama], 2008	1.31 (1.05, 1.64)
**Non-CVD mortality**	SD	Kikuya [Ohasama], 2008	1.18 (1.04, 1.34)	SD	Kikuya [Ohasama], 2008	1.07 (0.94, 1.22)	SD	Kikuya [Ohasama], 2008	1.15 (1.01, 1.31)
**Cerebral infarction mortality**	SD	Hashimoto [Ohasama], 2012	1.88 (1.31, 2.69)	SD	Kikuya [Ohasama], 2008	1.42 (1.08, 1.86)	SD	Kikuya [Ohasama], 2008	1.47 (1.11, 1.95)
**CVD events**	SD	Johansson [Health 2000], 2012	1.17 (1.02, 1.34)	SD	Johansson [Health 2000], 2012	1.08 (0.93, 1.26)	SD	Johansson [Health 2000], 2012	1.06 (0.93, 1.22)
**Stroke events**	VIM	Asayama [Ohasama], 2013	1.14 (1.00, 1.30)	VIM	Asayama [Ohasama], 2013	1.06 (0.93, 1.21)	-	-	-

Supplementary data – short term variability in ambulatory BP (daytime)

Figure e5

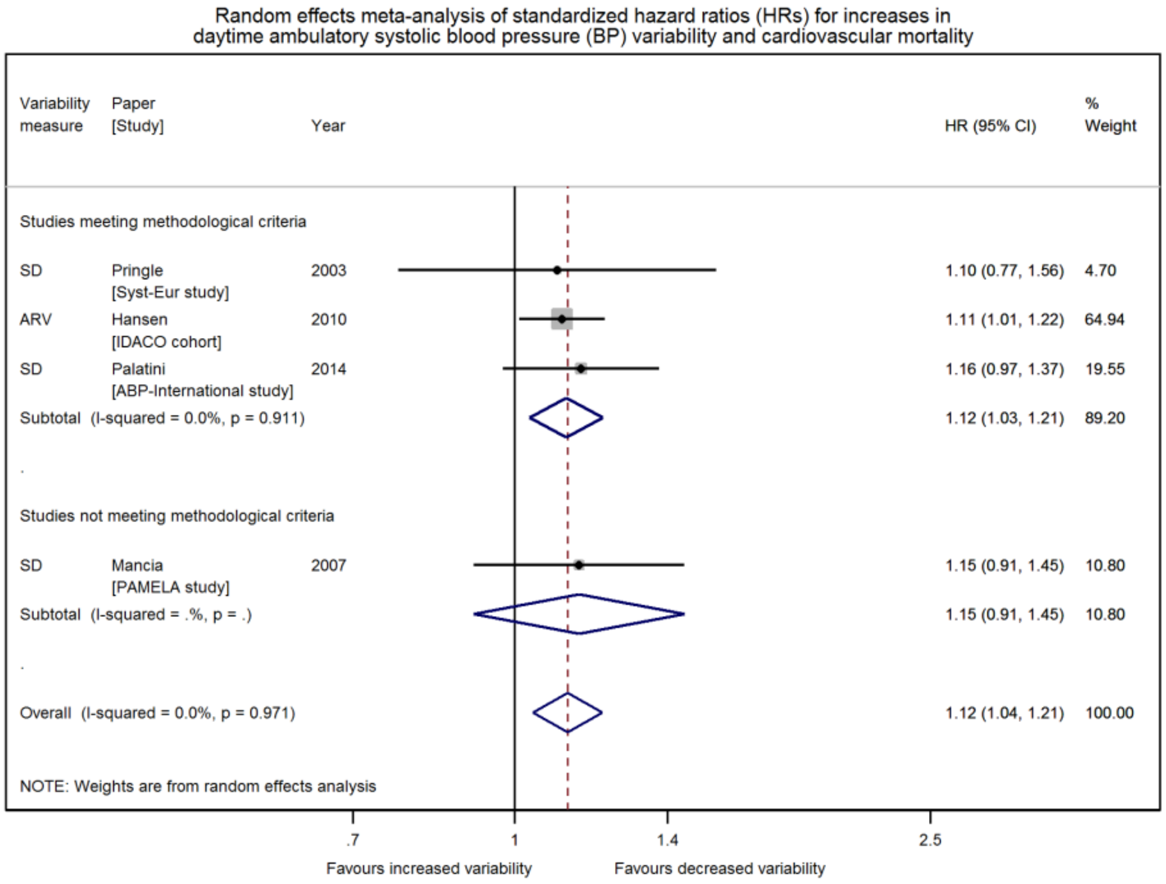

SD: Standard deviation, ARV: Average real variability

Figure e6

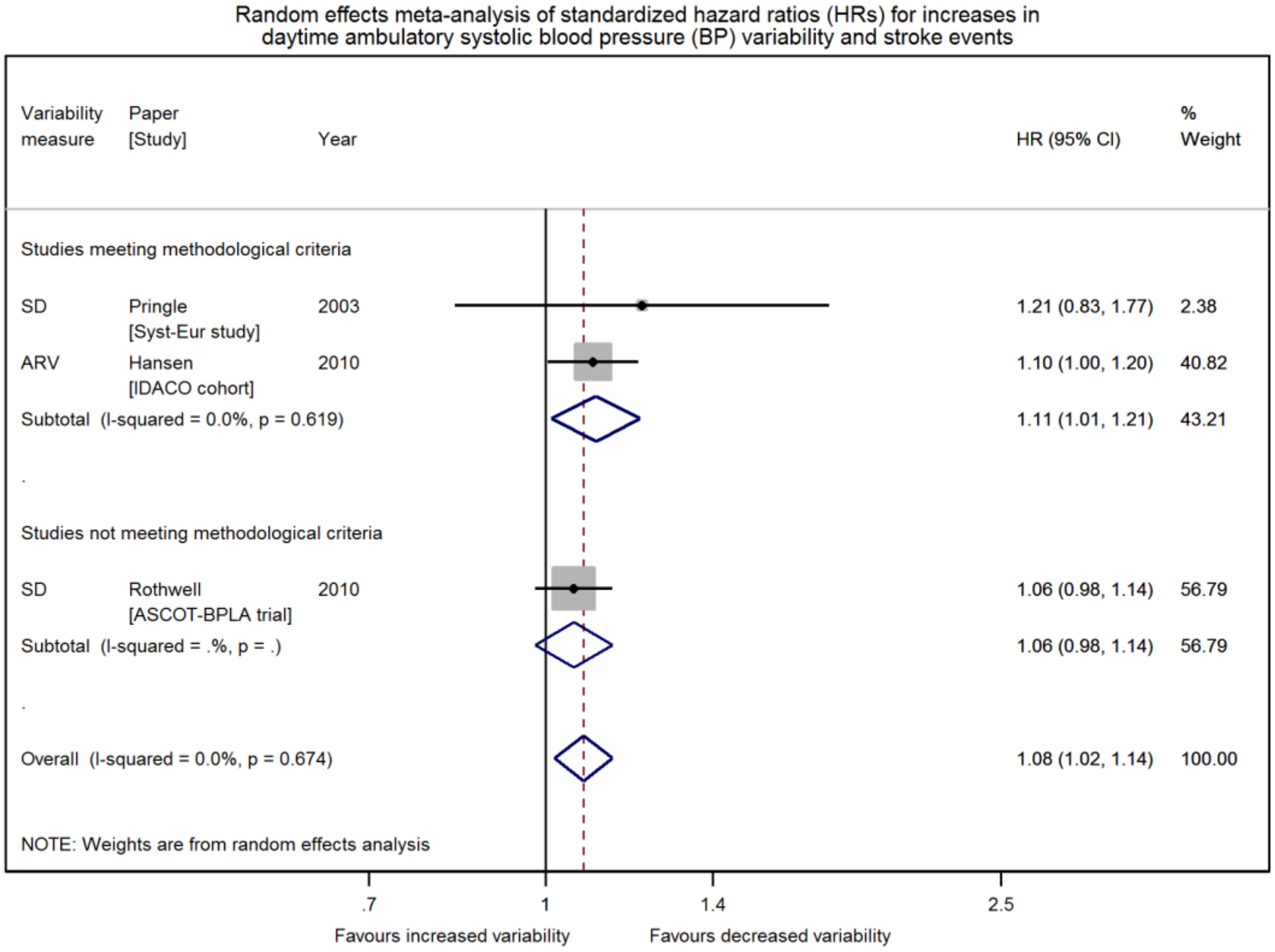

SD: Standard deviation, ARV: Average real variability

Figure e7

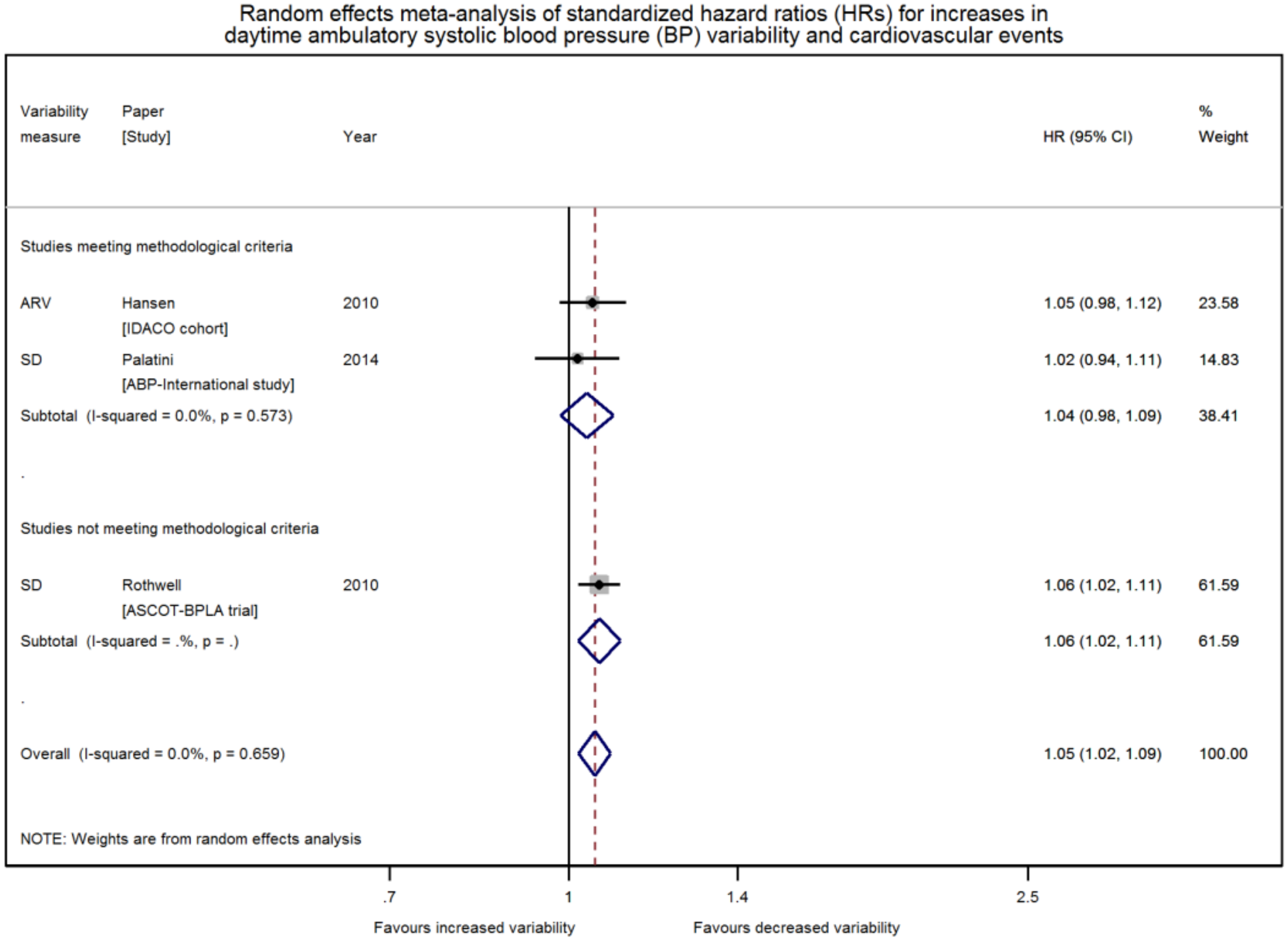

SD: Standard deviation, ARV: Average real variability

Figure e8

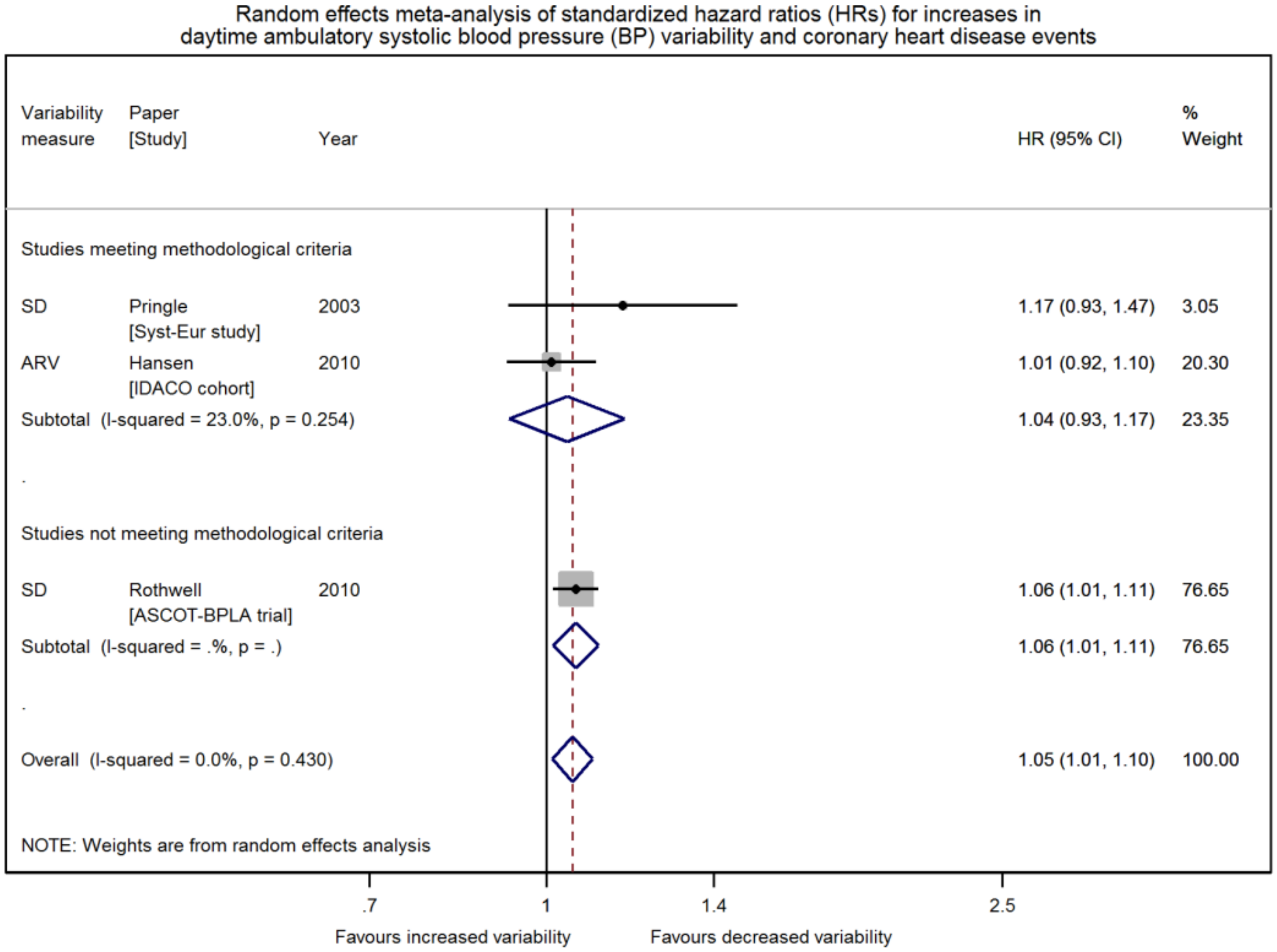

SD: Standard deviation, ARV: Average real variability

Supplementary data – short term variability in ambulatory BP (night-time)

Figure e9

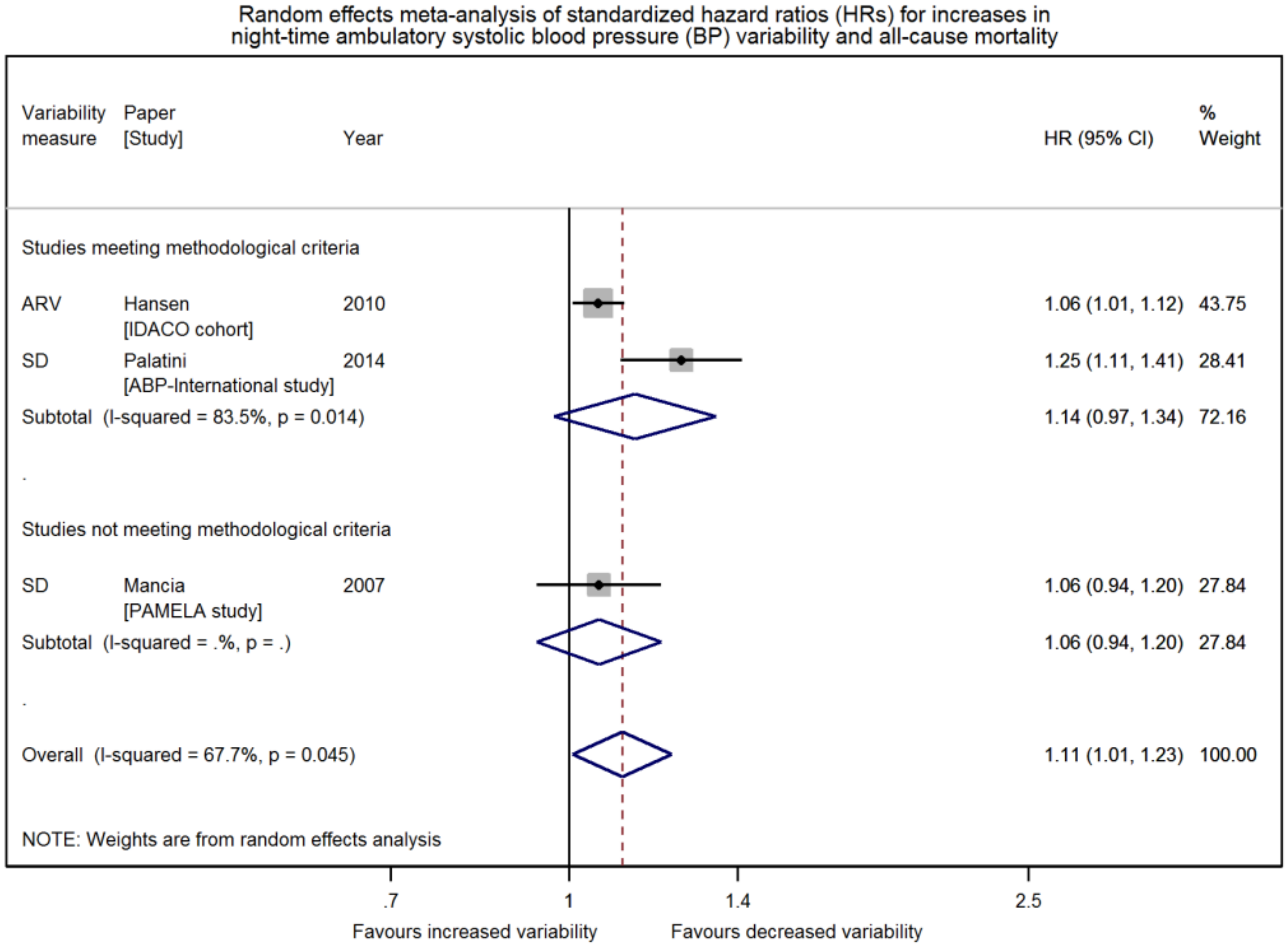

ARV: Average real variability, SD: Standard deviation

Figure e10

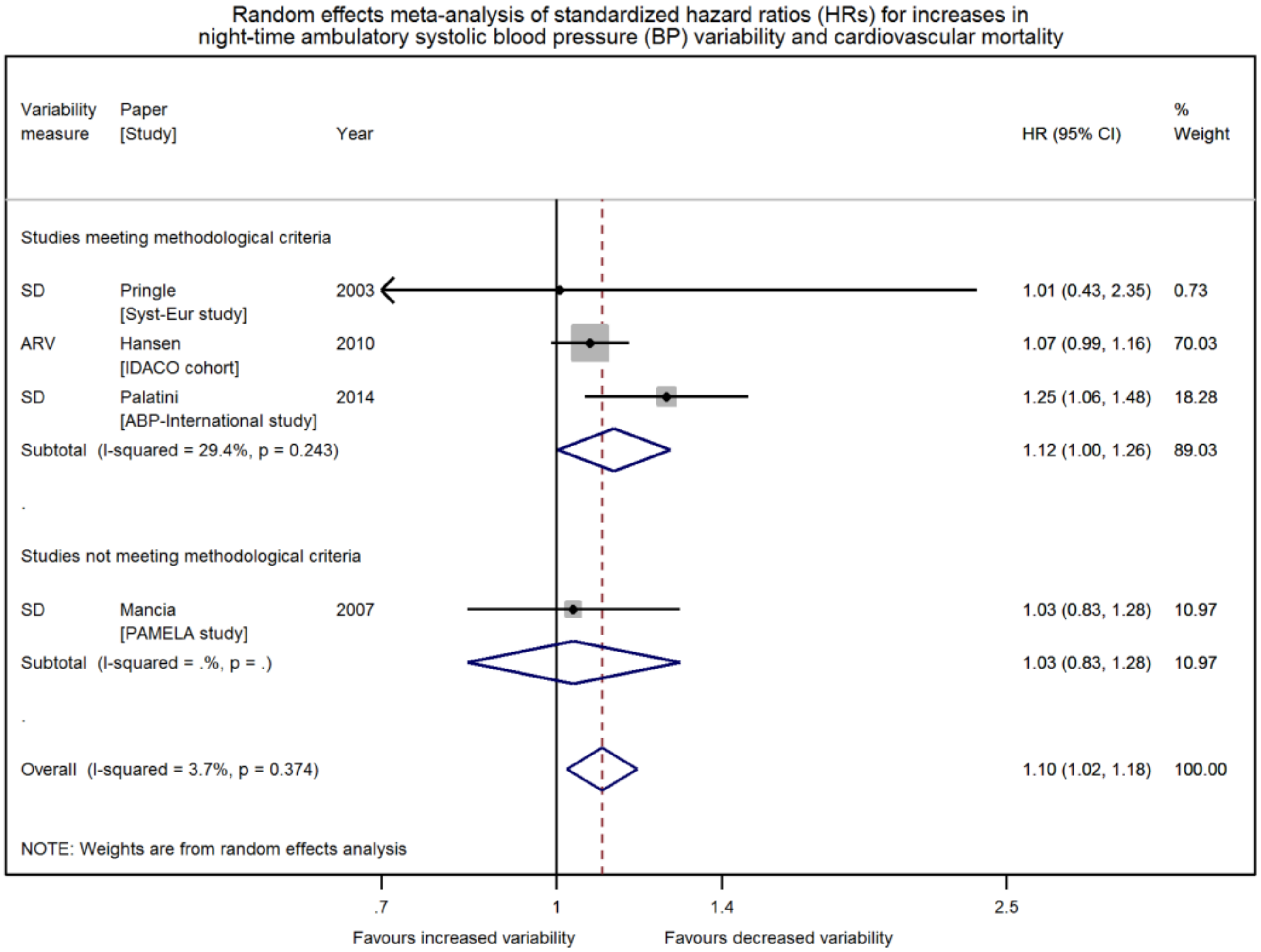

SD: Standard deviation, ARV: Average real variability

Figure e11

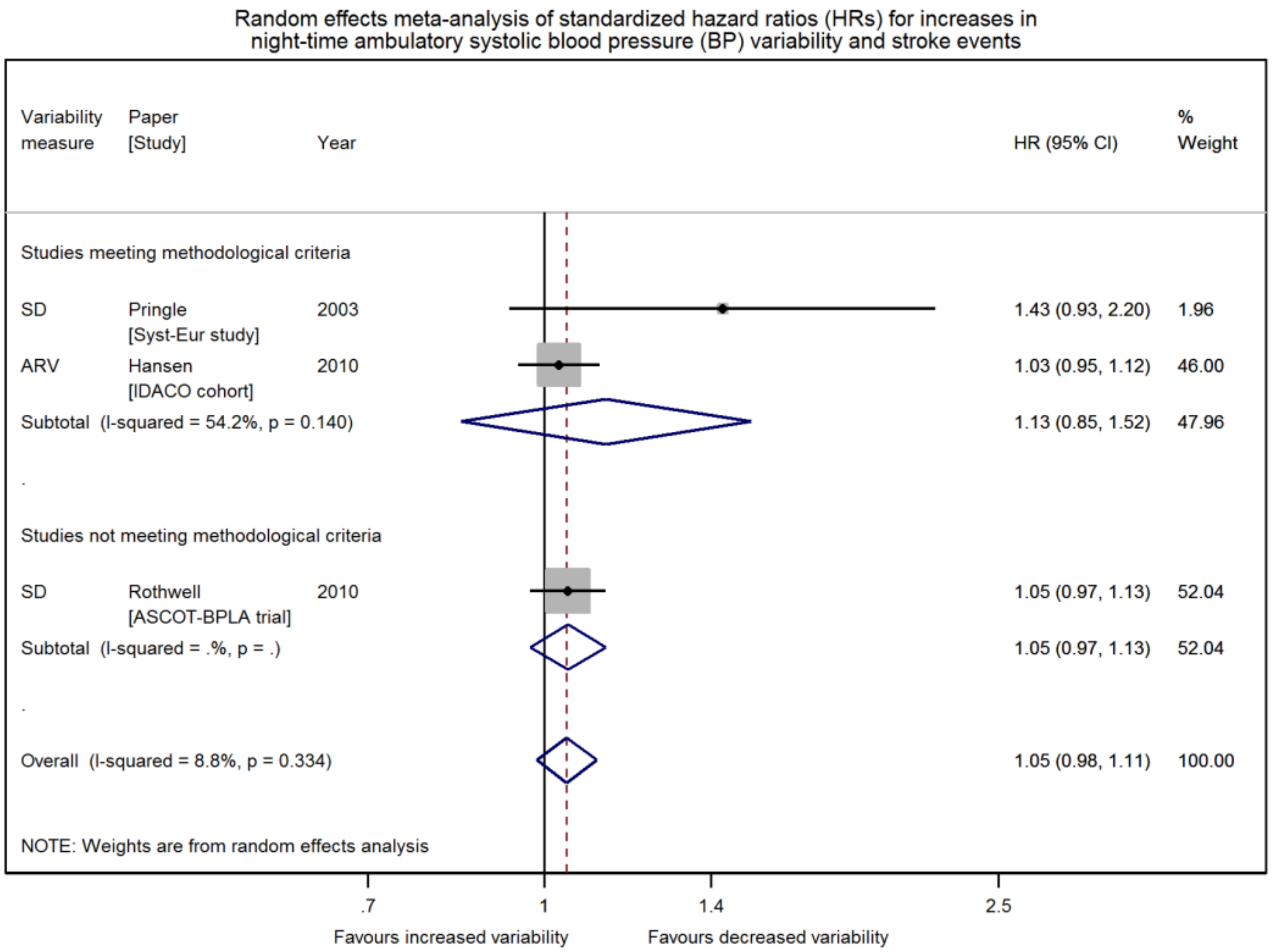

SD: Standard deviation, ARV: Average real variability

Figure e12

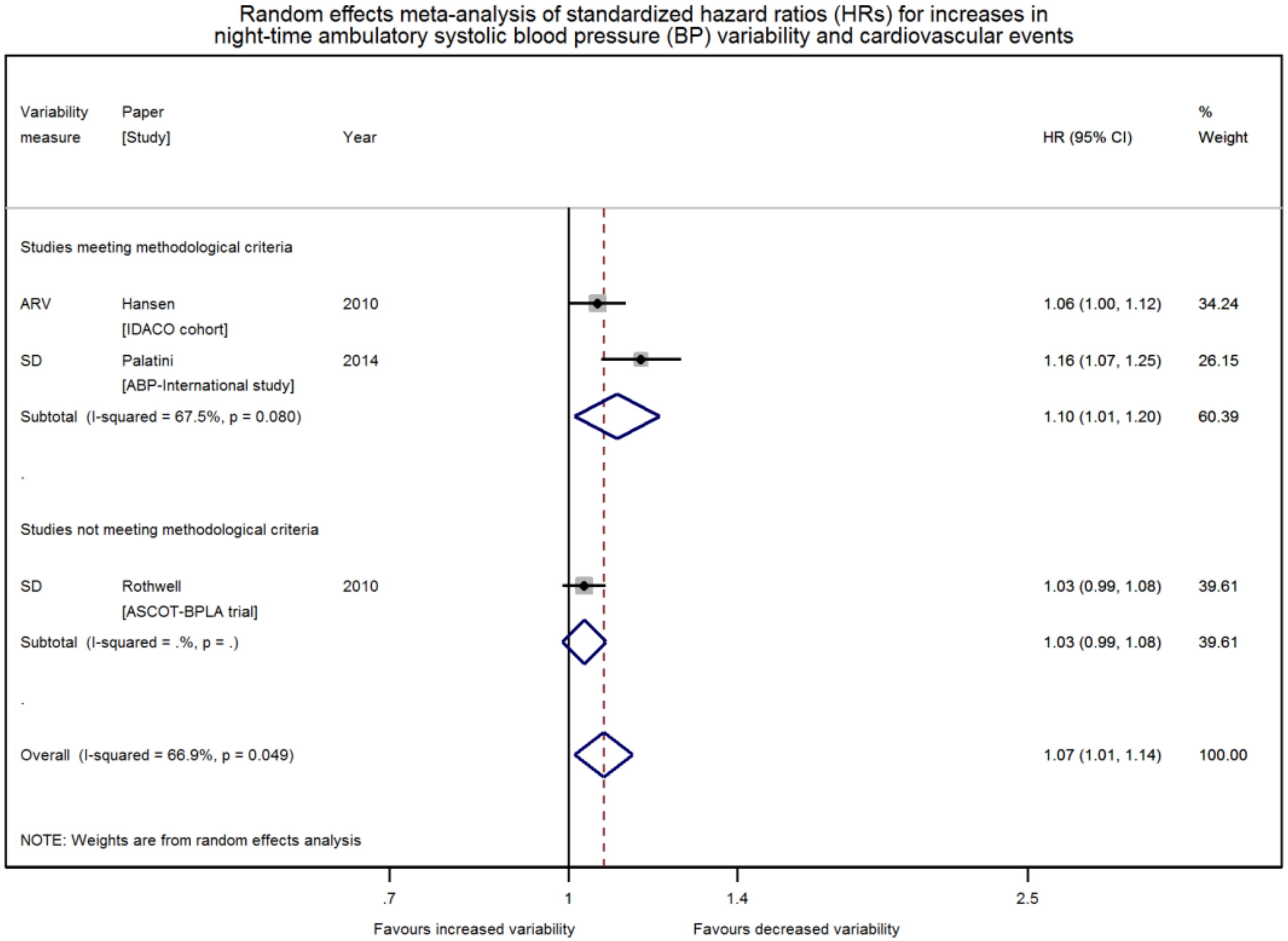

SD: Standard deviation, ARV: Average real variability

Figure e13

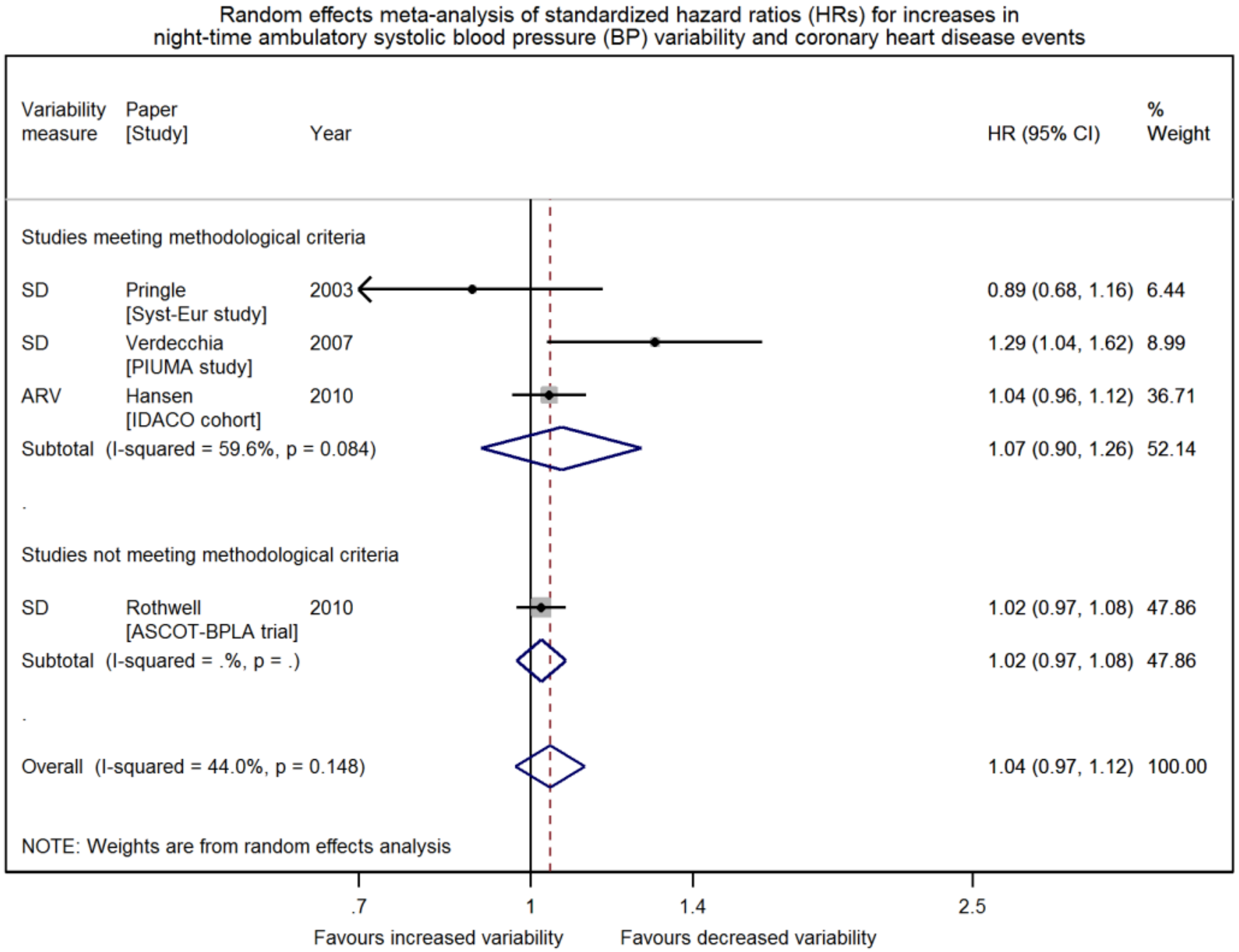

SD: Standard deviation, ARV: Average real variability

Supplementary data – short term variability in ambulatory BP (24-hour)

Figure e14

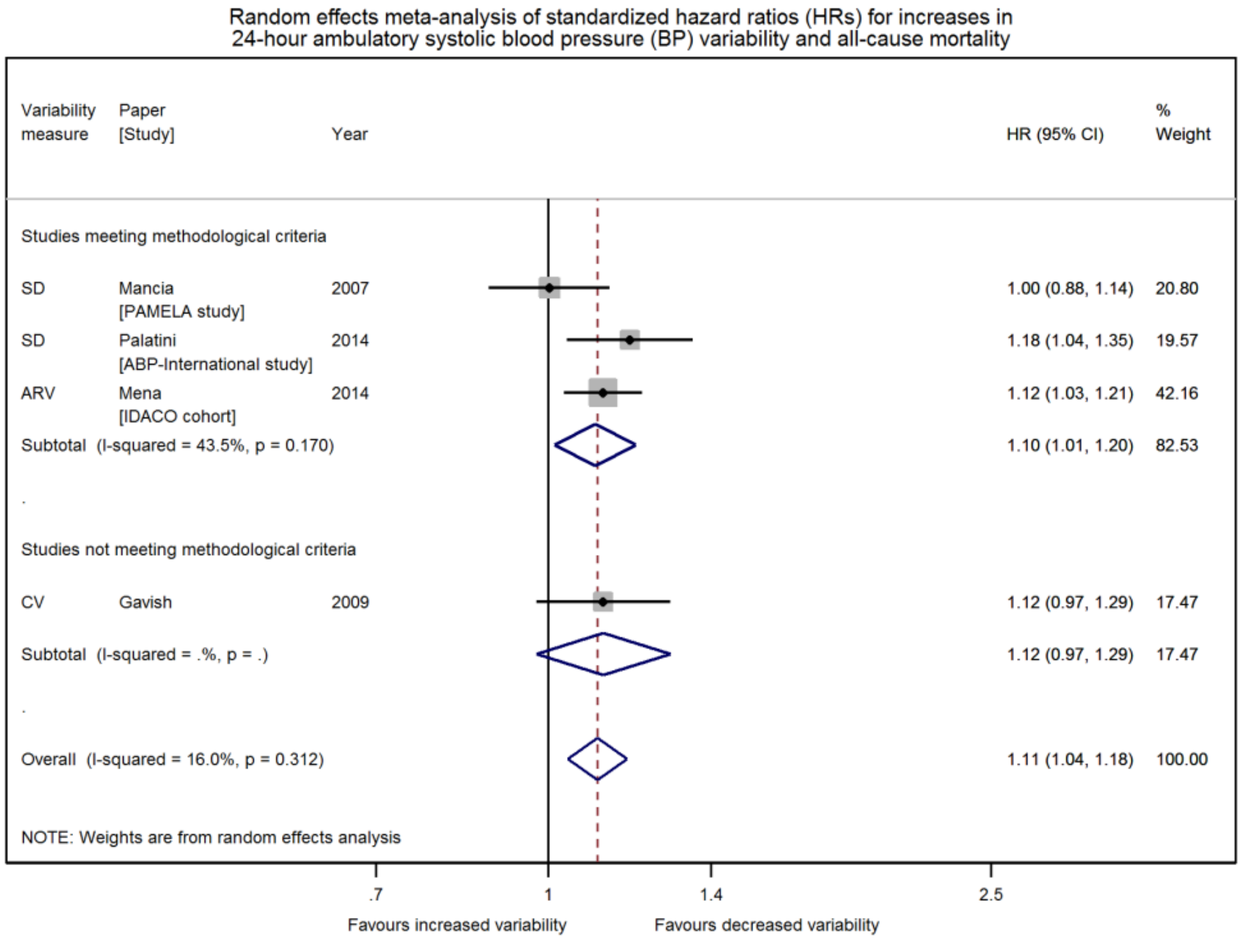

SD: Standard deviation, ARV: Average real variability, CV: Coefficient of variation

Figure e15

Random effects meta-analysis of standardized hazard ratios (HRs) for increases in
24-hour ambulatory systolic blood pressure (BP) variability and cardiovascular mortality

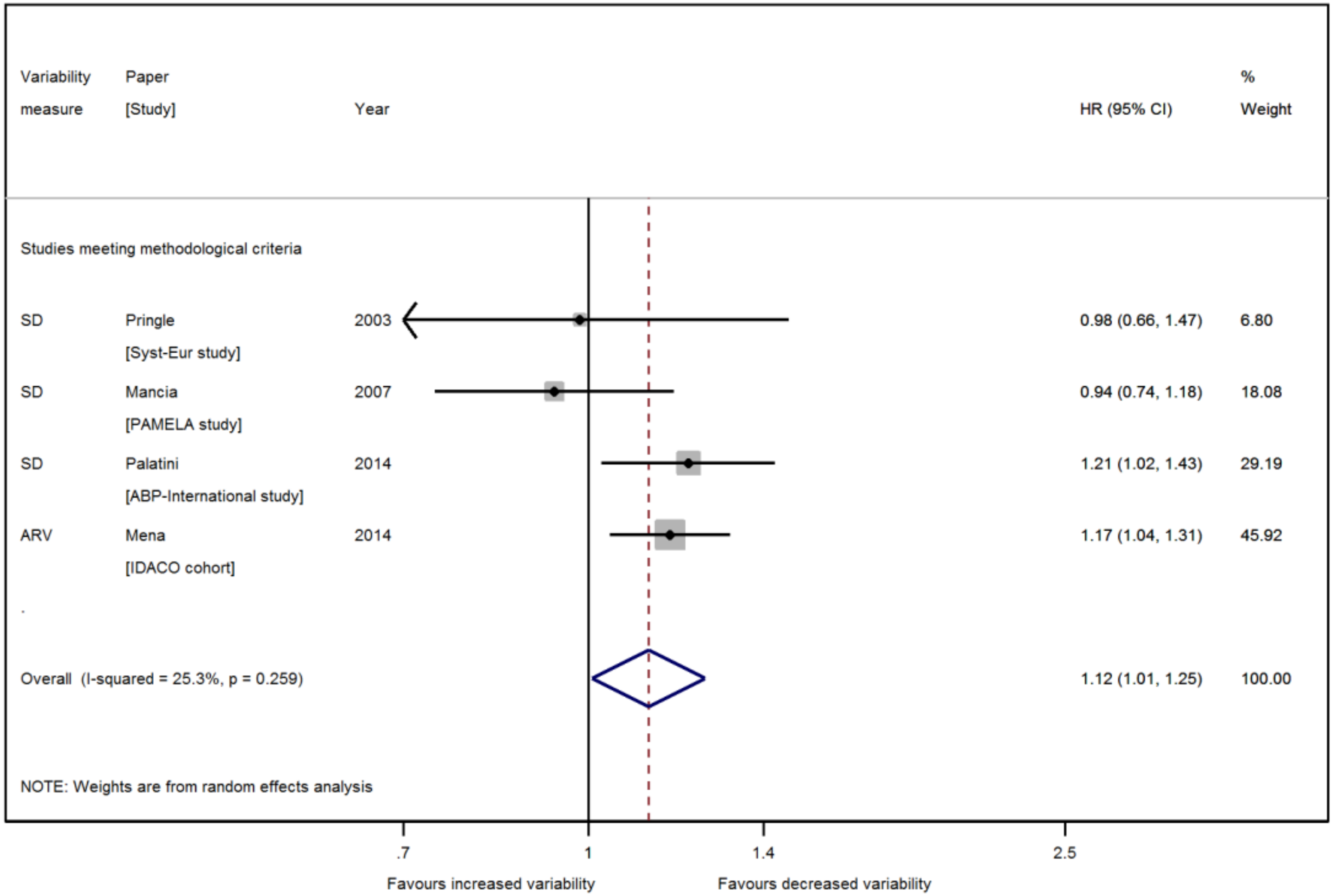

SD: Standard deviation, ARV: Average real variability

Figure e16

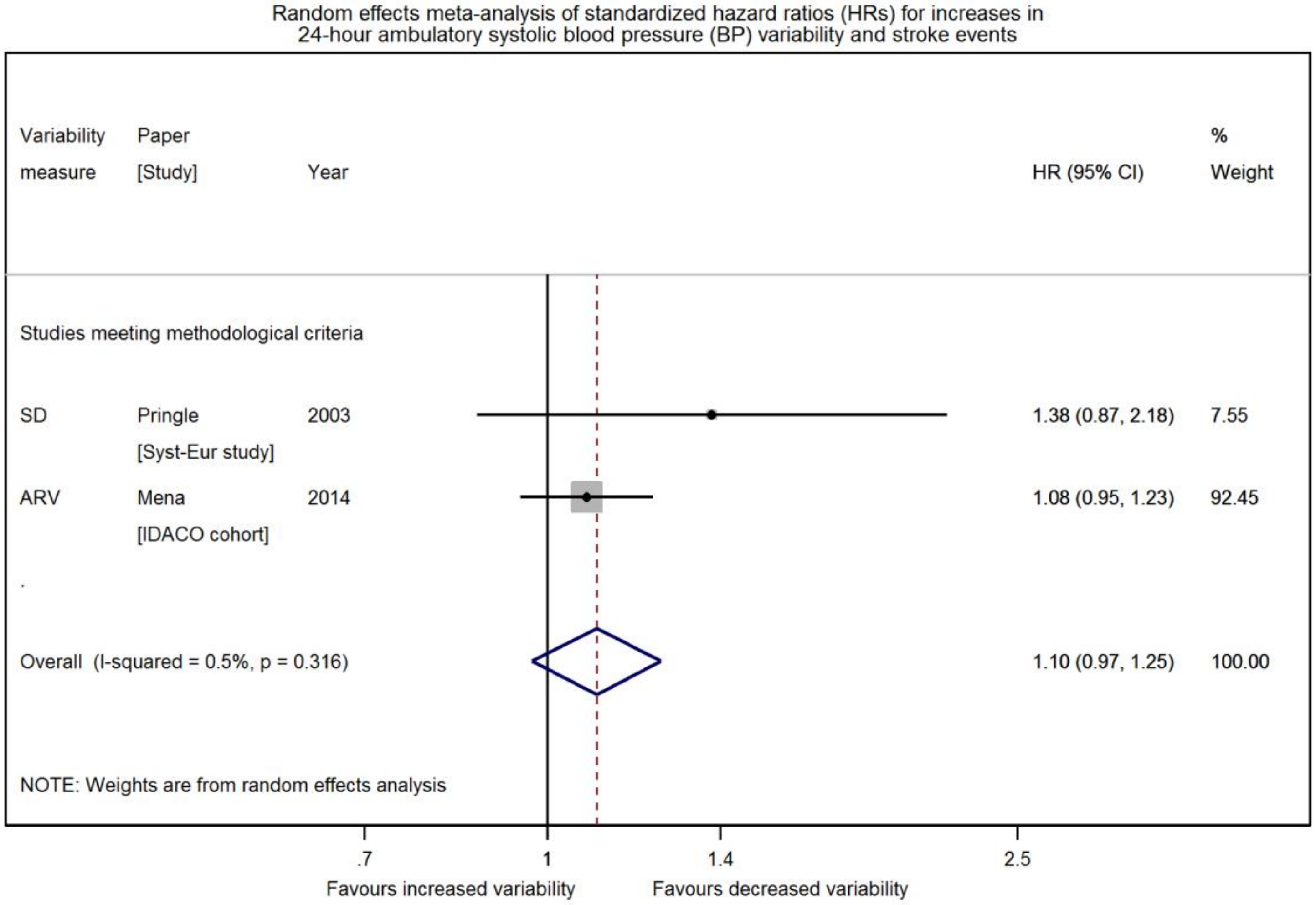

SD: Standard deviation, ARV: Average real variability

Figure e17

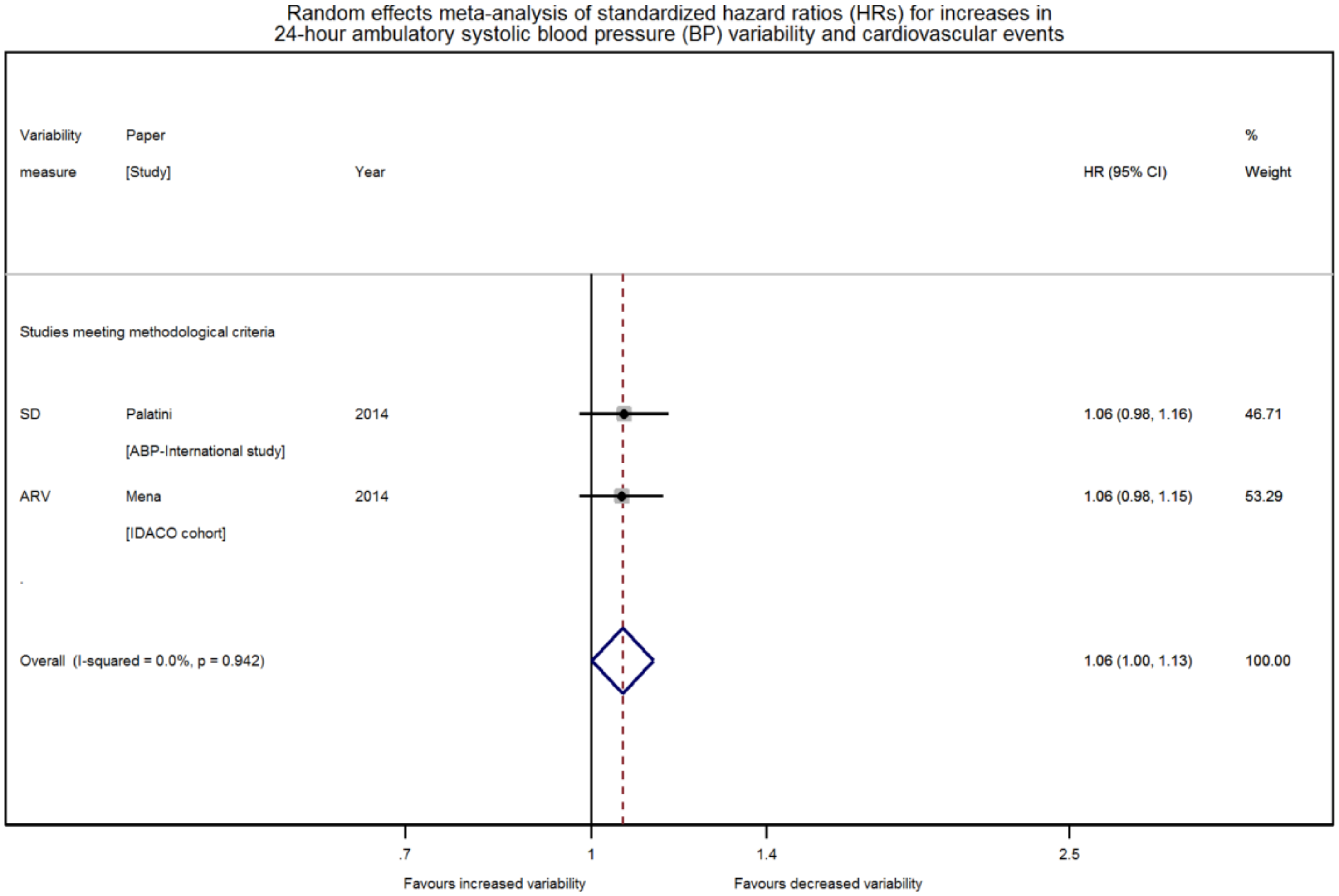

ARV: Average real variability

Figure e18

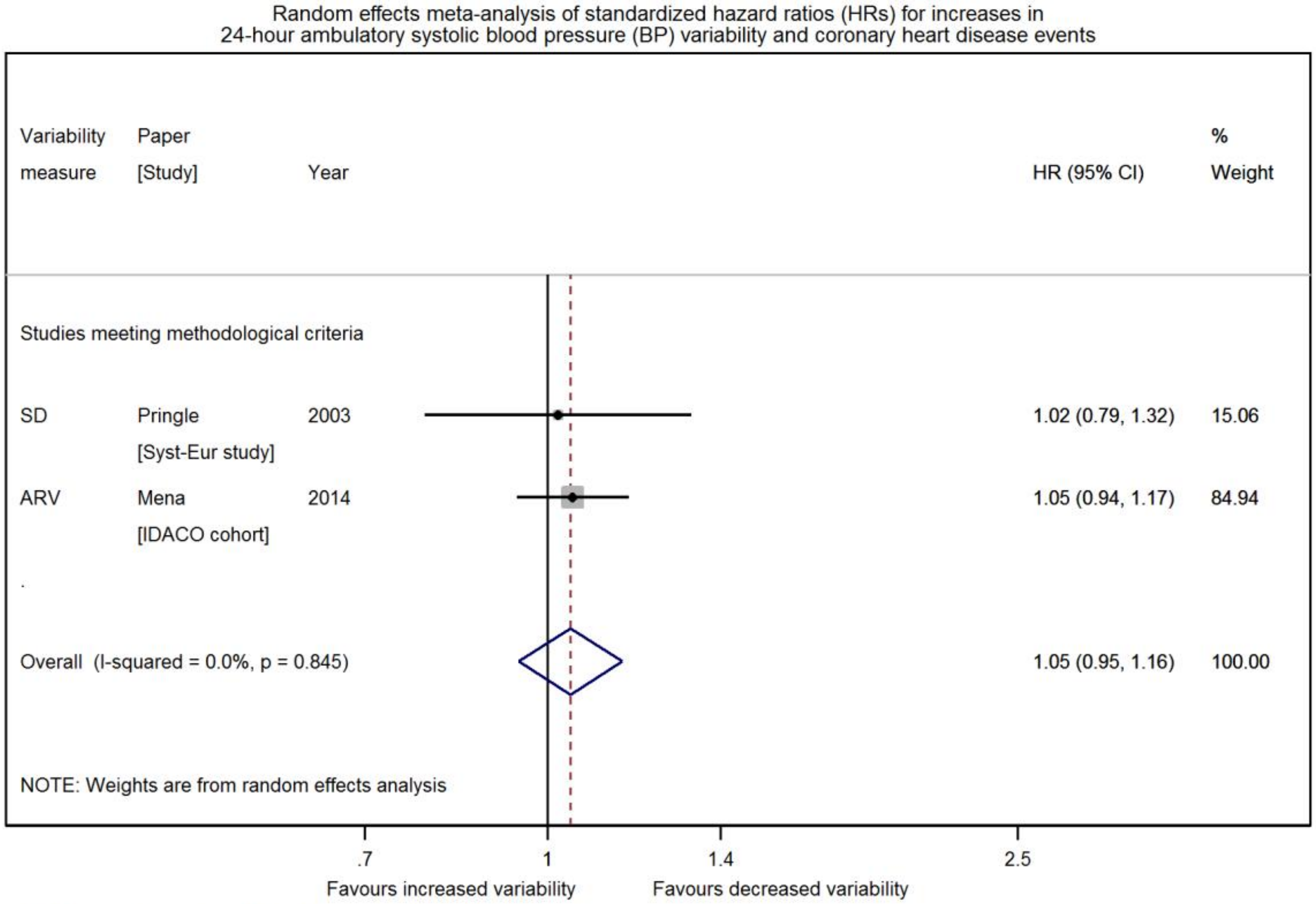

SD: Standard deviation, ARV: Average real variability
